# Supplementary figures and images for: Artificial neural networks for model identification and parameter estimation in computational cognitive models
Source: PLoS Comput Biol. 2024 May 15;20(5):e1012119. doi: 10.1371/journal.pcbi.1012119 (PMC11132492; doi:10.1371/journal.pcbi.1012119)

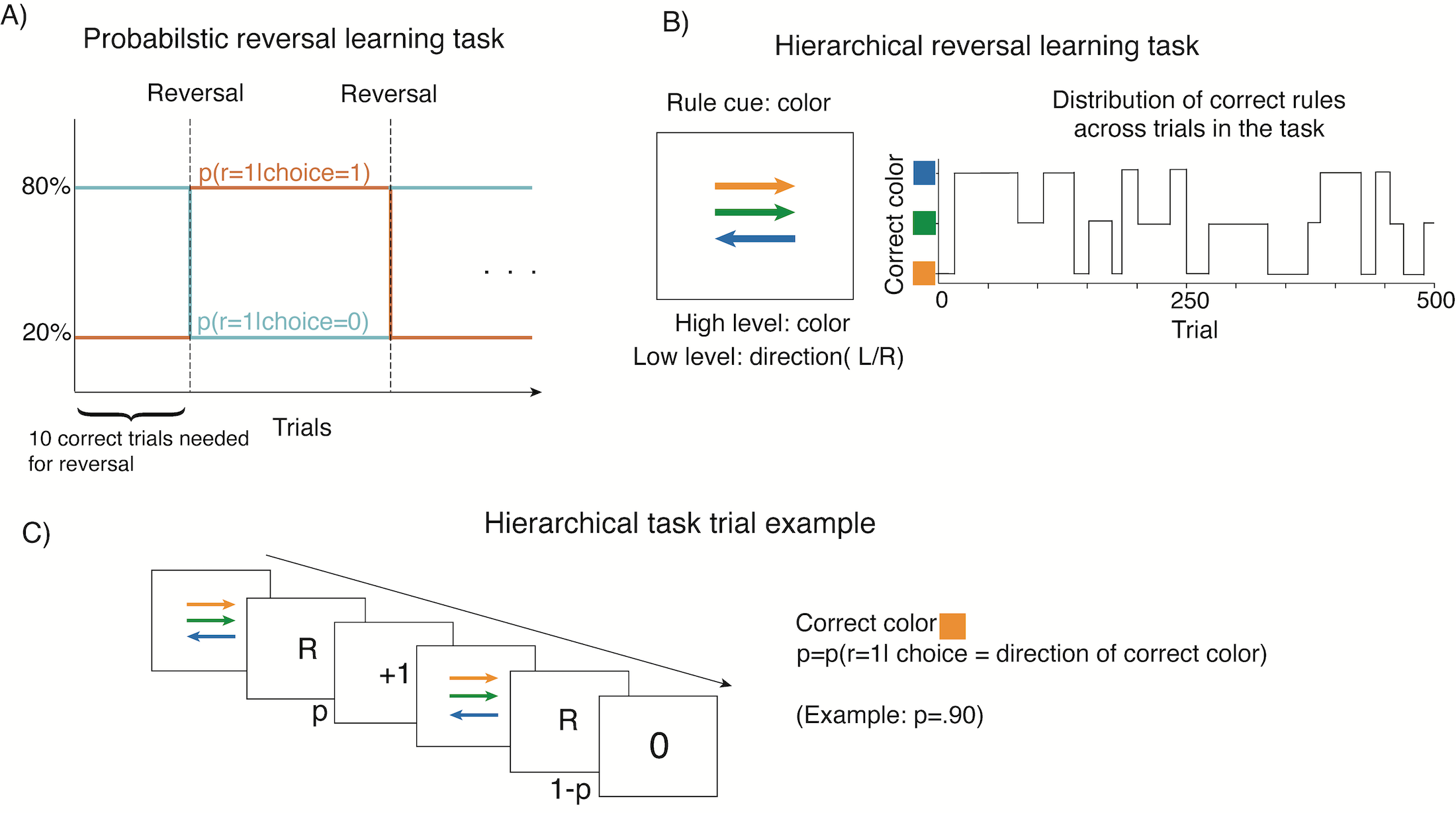

Supplement: S1 Fig — A) Probabilistic Reversal Learning task. We simulated artificial agents using cognitive models of behavior on a Probabilistic reversal learning (PRL) task, which provides a dynamic context for studying reward-driven learning. In this task, an agent chooses between two actions, where one of the actions gets rewarded with higher probability (p(r) =.80) and one with lower (1 − p). After a certain number of correct trials, the reward probabilities of the two actions reverse. The task provides an opportunity to observe how agents update their model of the task (e.g. correct actions) based on observed feedback. B) Hierarchical reinforcement learning task. In this task, three differently colored arrows represent three potential rules an agent can follow when selecting one of the two actions (left/right) corresponding to the side the chosen arrow is pointing at. Selecting a side consistent with correct arrow is rewarded with probability p =.90. Correct arrow switches after a certain number of trials. The task provides a possibility to examine how following latent rules may shape agents’ learning behavior. (TIF) [file pcbi.1012119.s001.tif]

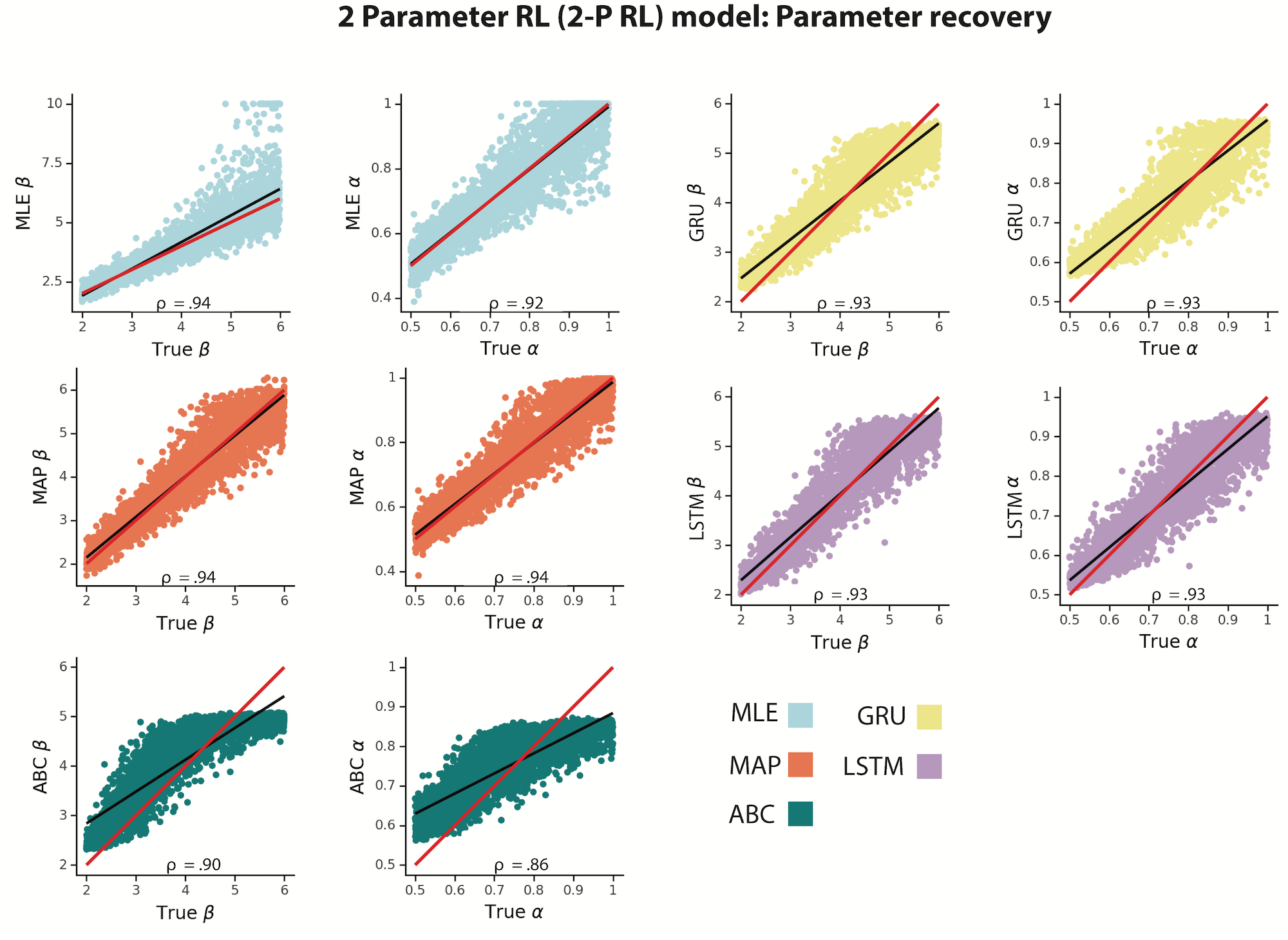

Supplement: S2 Fig — ρ corresponds to Spearman correlation coefficient. The red line represents a unity line (x = y), and the black line represents a least squares regression line. (TIF) [file pcbi.1012119.s002.tif]

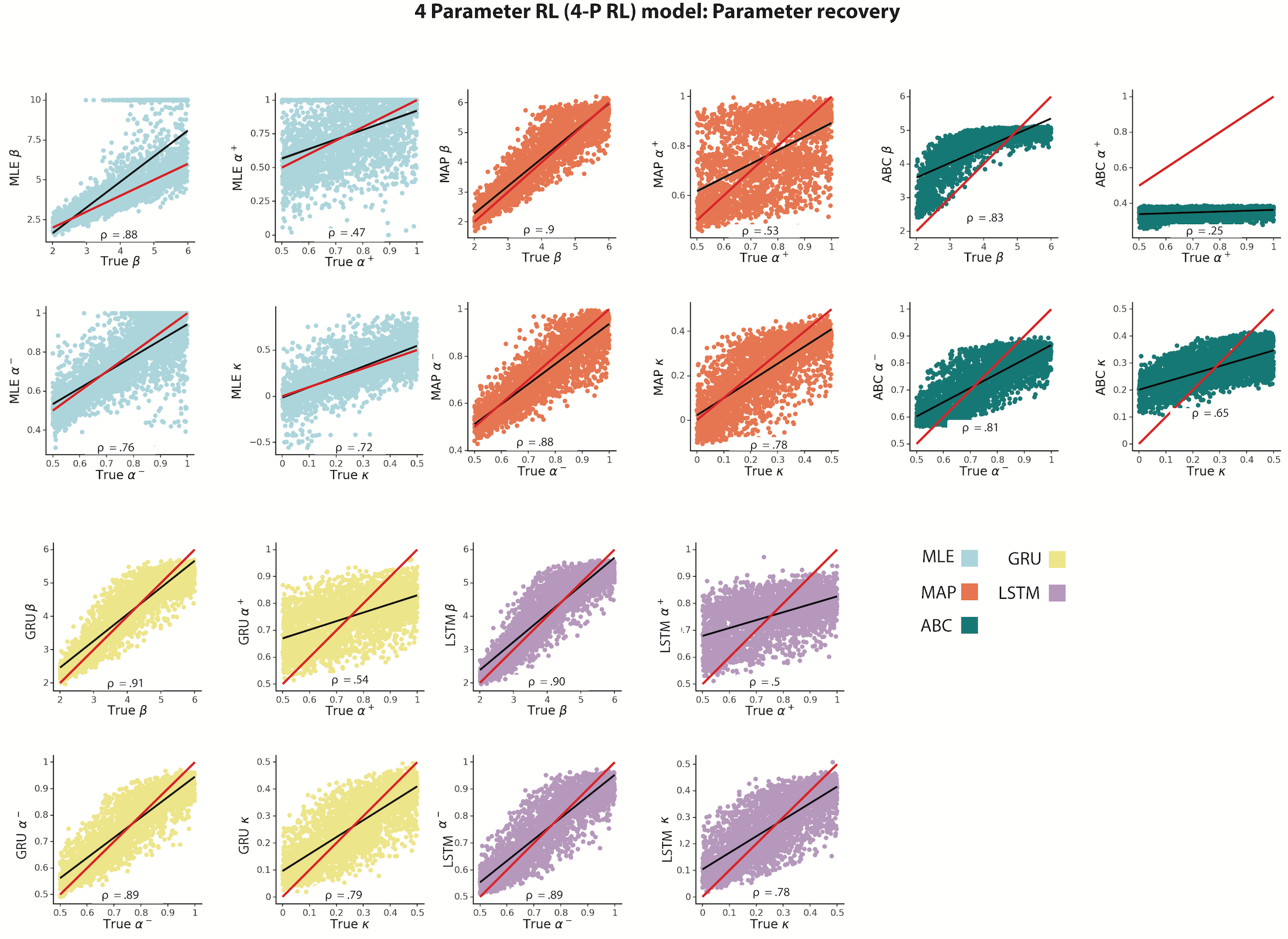

Supplement: S3 Fig — ρ corresponds to Spearman correlation coefficient. The red line represents a unity line (x = y), and the black line represents a least squares regression line. (TIF) [file pcbi.1012119.s003.tif]

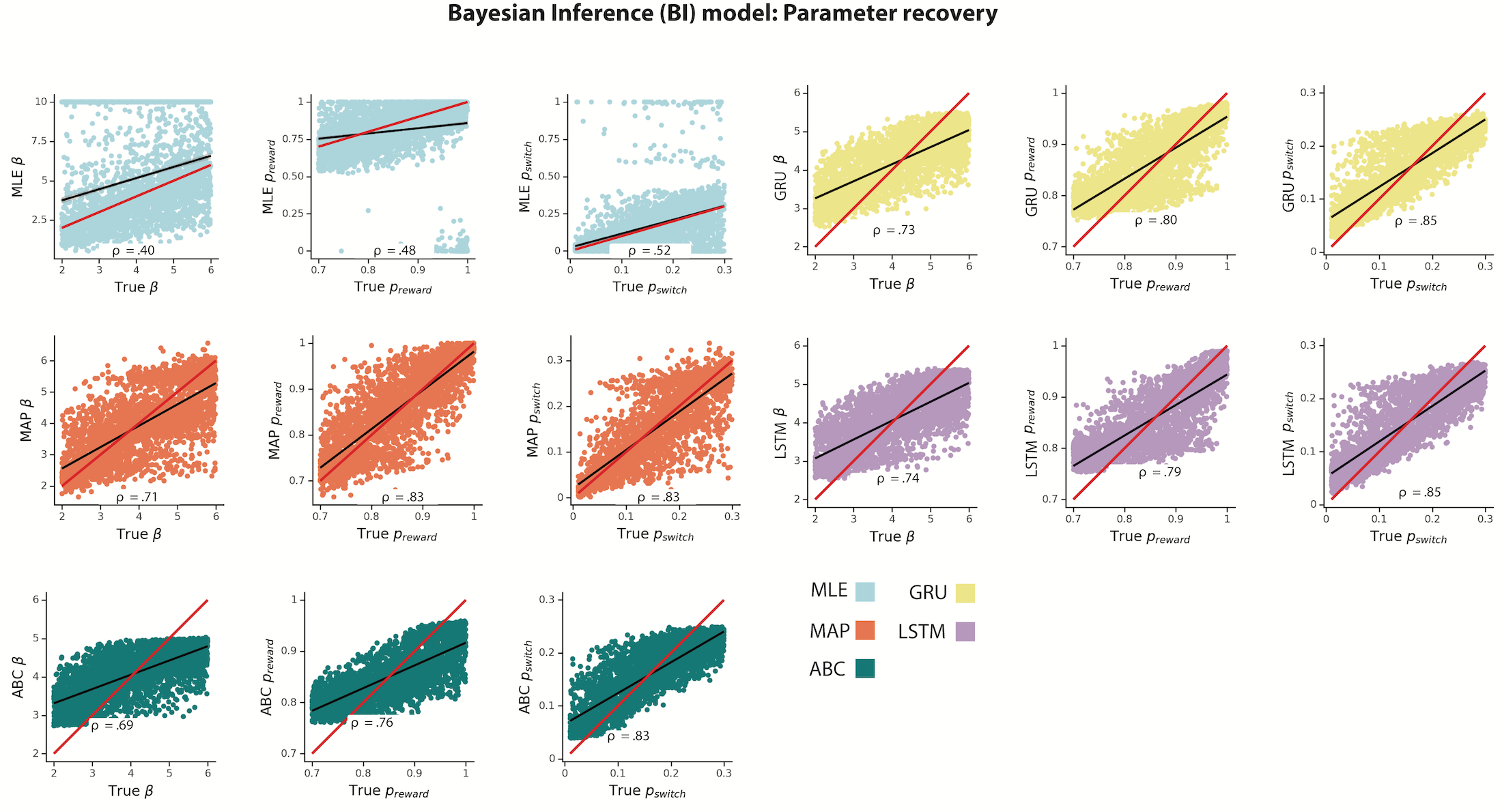

Supplement: S4 Fig — ρ corresponds to Spearman correlation coefficient. The red line represents a unity line (x = y), and the black line represents a least squares regression line. (TIF) [file pcbi.1012119.s004.tif]

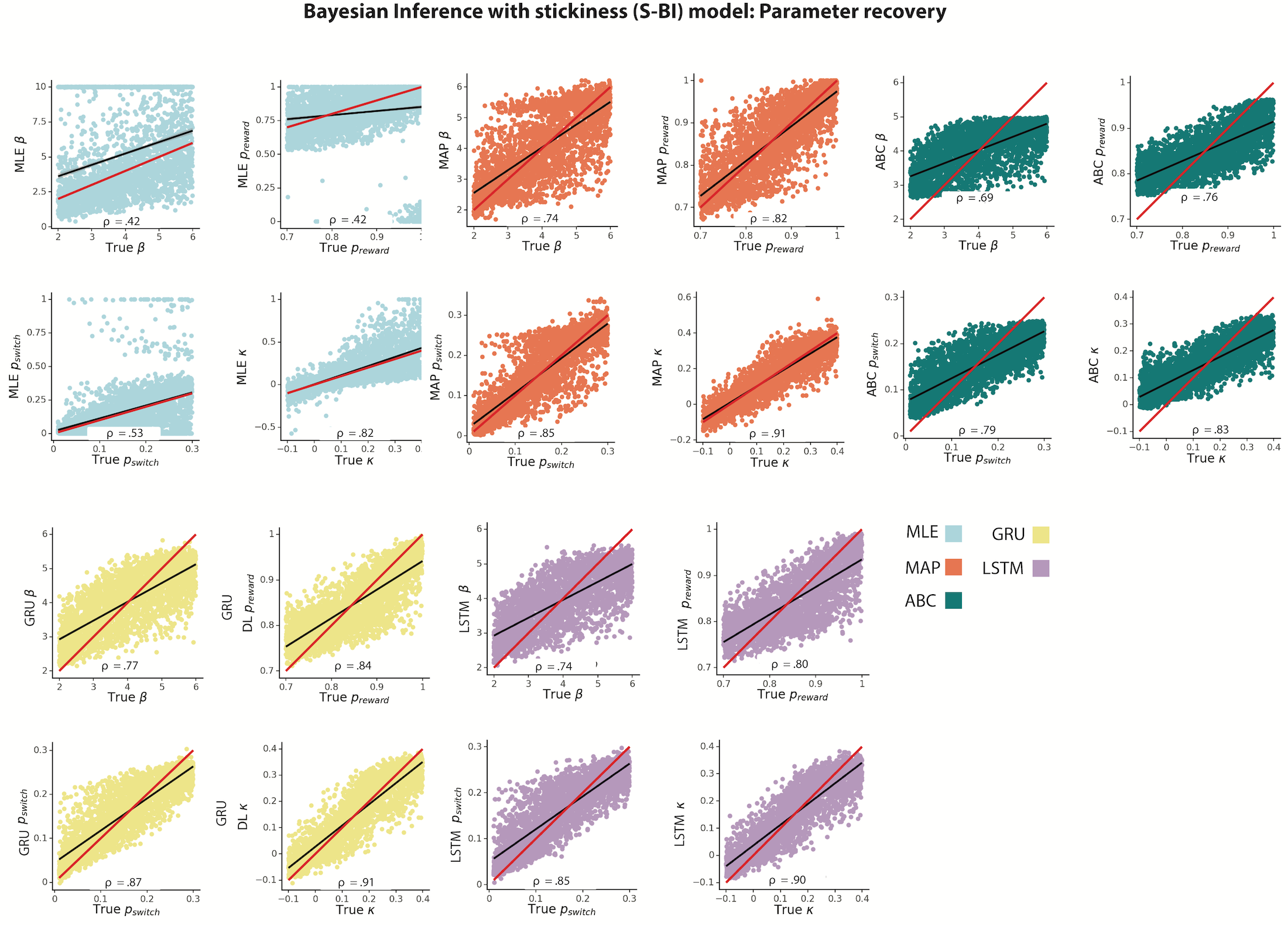

Supplement: S5 Fig — ρ corresponds to Spearman correlation coefficient. The red line represents a unity line (x = y), and the black line represents a least squares regression line. (TIF) [file pcbi.1012119.s005.tif]

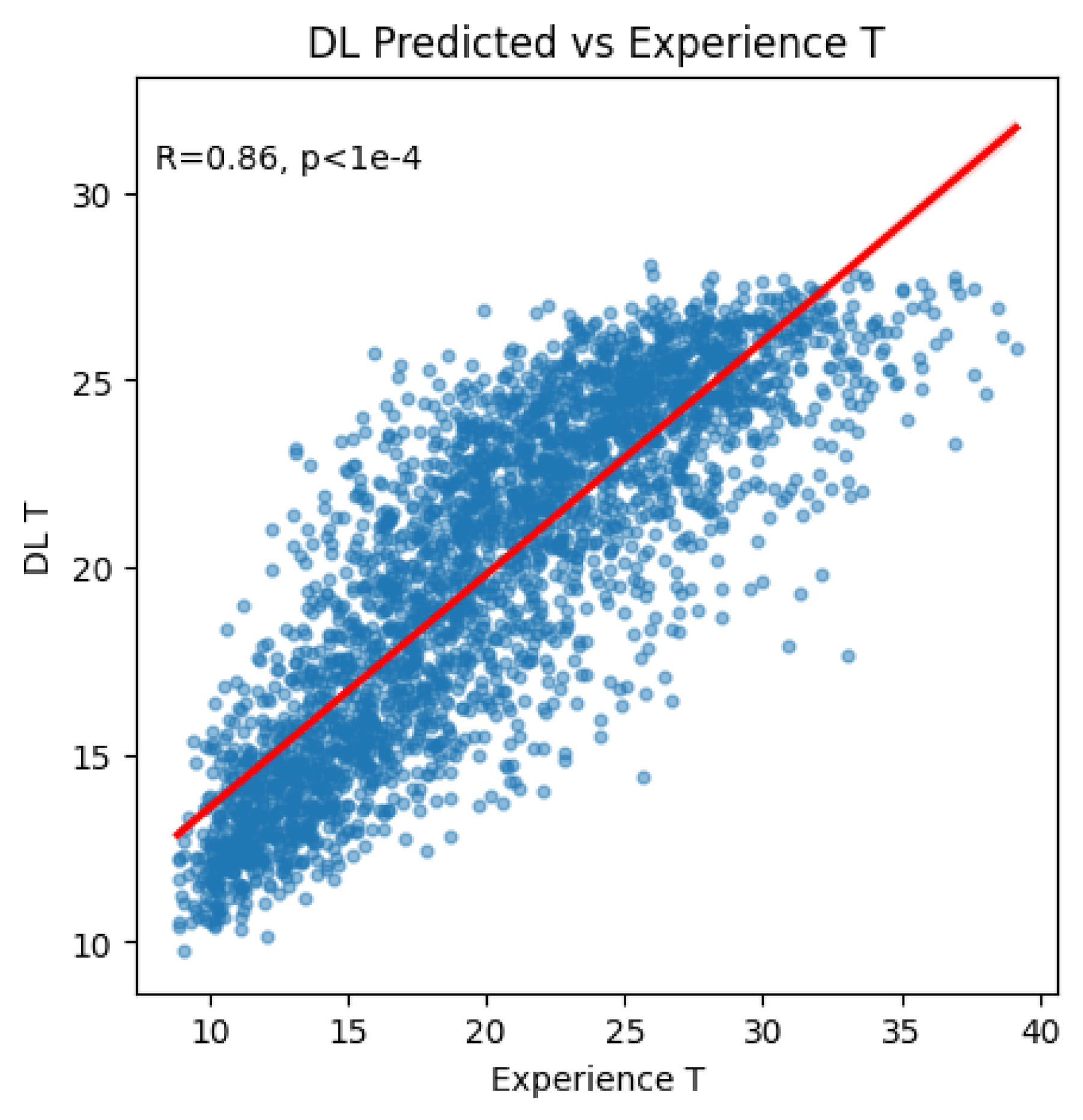

Supplement: S6 Fig — Correlation between the average experienced time intervals in the attentive state and the τ parameter in RL-LAS model that captures transition between disengaged/engaged attention states estimated by the ANN. (TIF) [file pcbi.1012119.s006.tif]

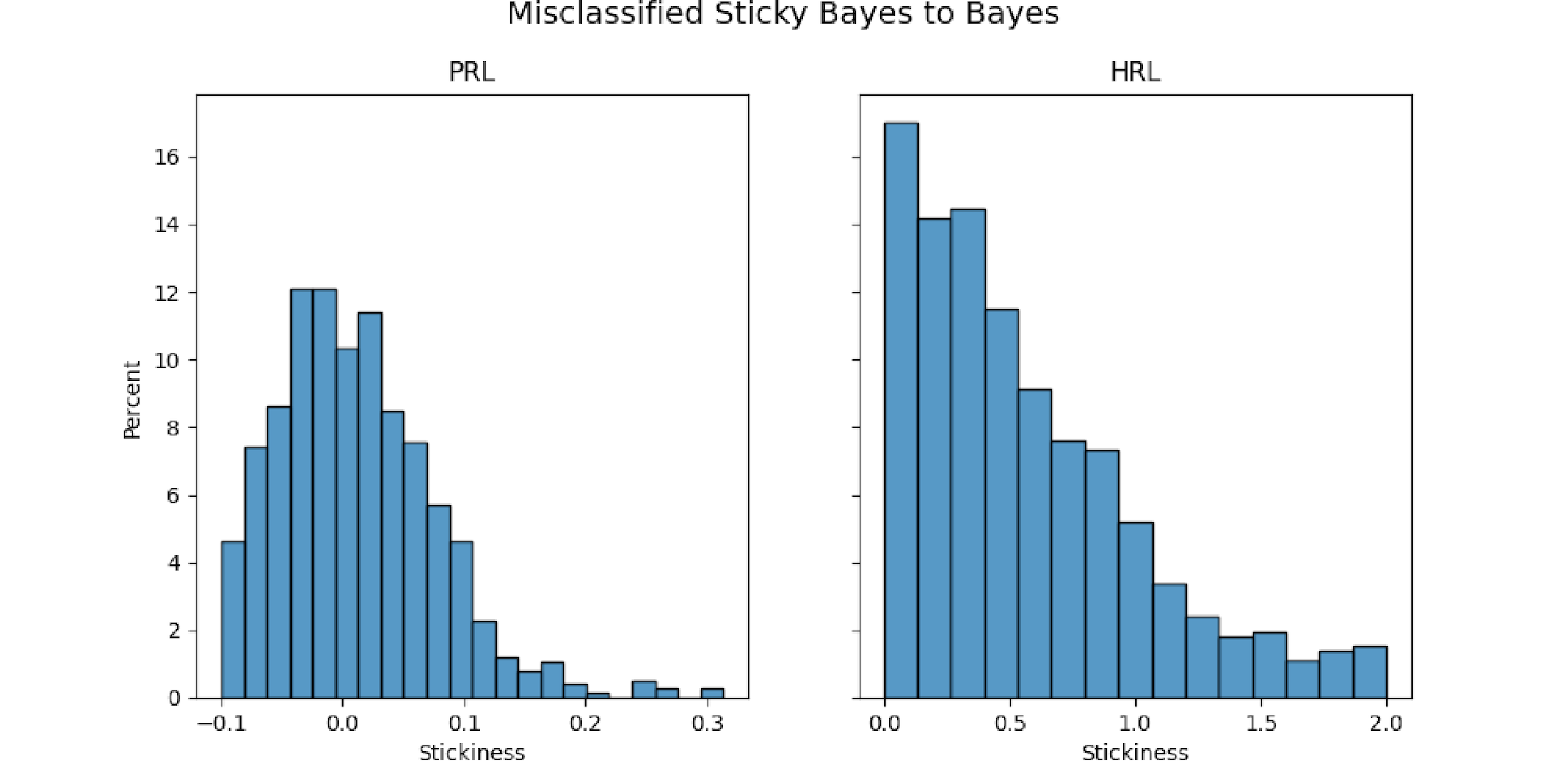

Supplement: S7 Fig — Misclassification of Bayes and sticky Bayes model is contingent on the value of the stickiness parameter κ. The misclassification percentage is higher at κ values closer to 0. (TIF) [file pcbi.1012119.s007.tif]

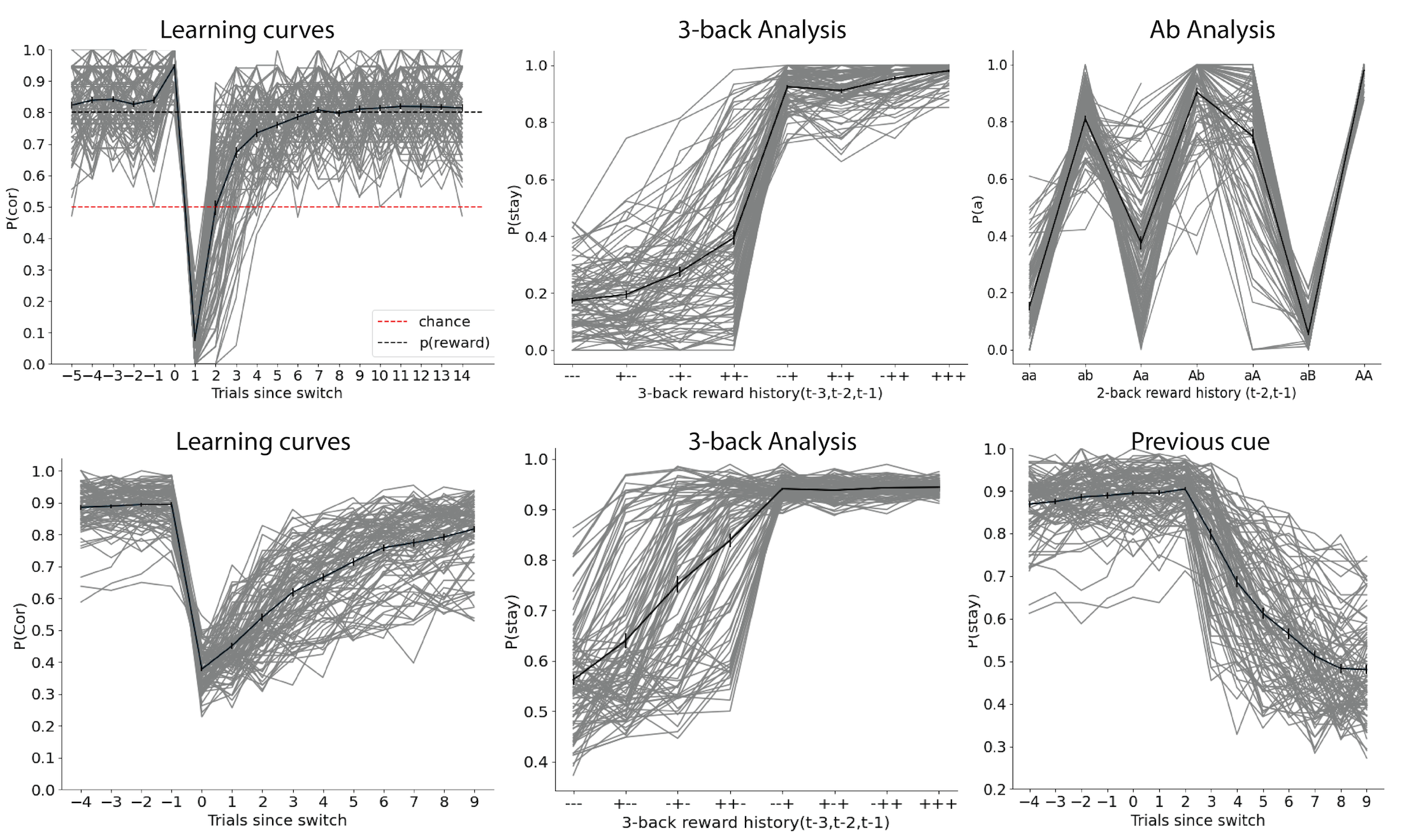

Supplement: S8 Fig — Top row shows summary statistics computed for all models simulated on a probabilistic reversal learning task; the figure only shows agents simulated using a 4-parameter RL model. The bottom row shows summary statistics computed for all models simulated on a hierarchical reversal learning task; the figure only shows performance of HRL model agents. Both rows depict 200 out of 3000 test set agents. Gray lines represent individual agents, and the black line represents an average across the agents. (TIF) [file pcbi.1012119.s008.tif]

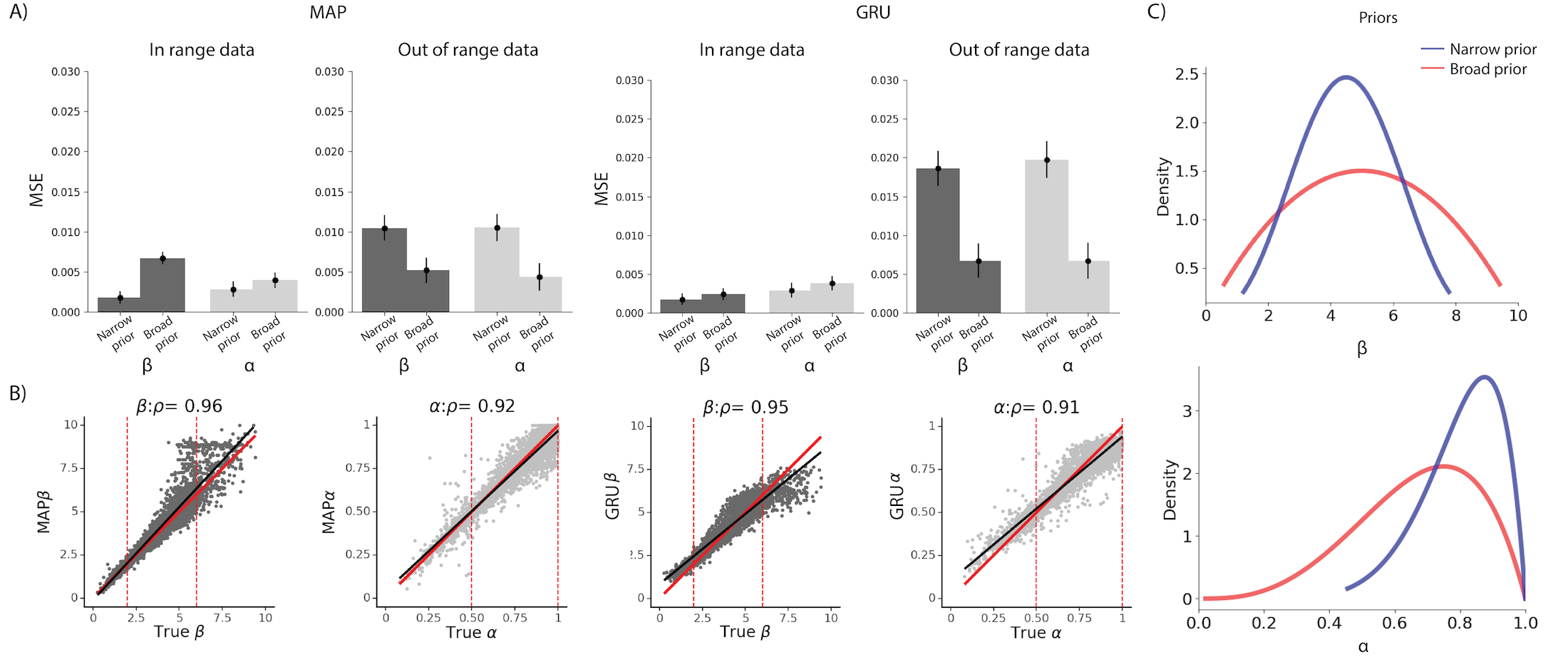

Supplement: S9 Fig — A) Applying too narrow a prior specification to the fitting procedure (prior in MAP, training samples in ANN) results in difficulty estimating out-of-range parameters for both MAP and ANN. Broader prior specification addresses this issue, with only a slight loss of precision in specific target ranges. Training the network with a broad range of parameters while oversampling parameters from regions of interest yields the most robust results. B) Visualization of fitting with MAP and ANN with a wide prior, tested on a full range/wide range data set—training the network with broader range while oversampling from the most plausible range yields less noisy performance in the range compared to MAP. Red lines delineate the range of the narrow prior, which corresponds to the main text results. C) The broad prior was designed by sampling from the full broader range (β ∈ [0, 10], α ∈ [0, 1]), with the constraint that 70% of samples are in the expected narrow range (β ∈ [2, 6], α ∈ [0.5, 1], and 30% outside). (TIF) [file pcbi.1012119.s009.tif]

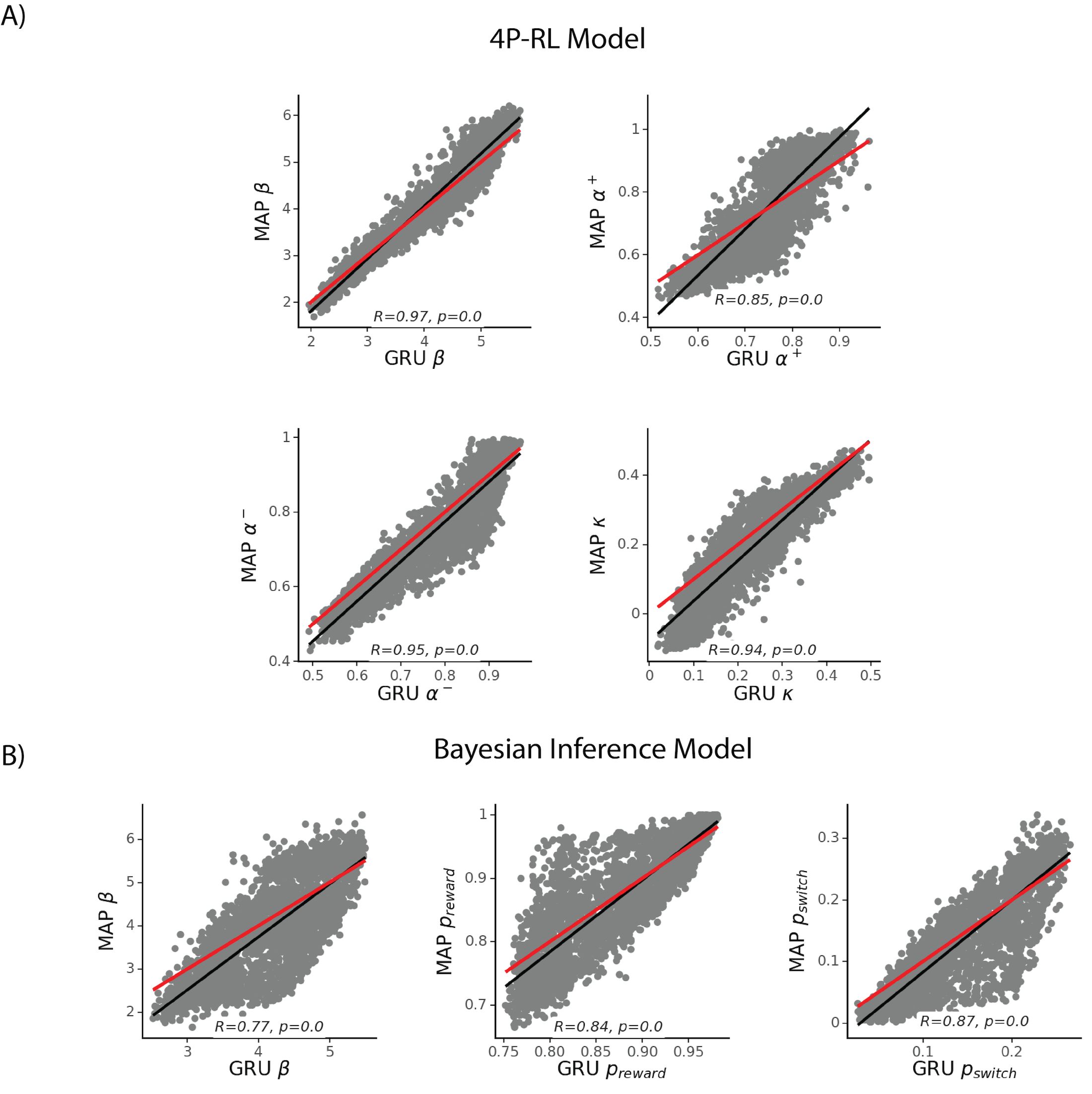

Supplement: S10 Fig — A) The correlation between parameter estimates in the 4P-RL model derived using MAP and ANN is high, and indeed stronger than the correlation between true and derived parameters (see Fig 2). This indicates that both methods systematically misidentify some parameters similarly, likely due to specific data patterns. B) The correlation between parameter estimates in the Bayesian inference model derived using MAP and ANN shows similar results. (TIF) [file pcbi.1012119.s010.tif]

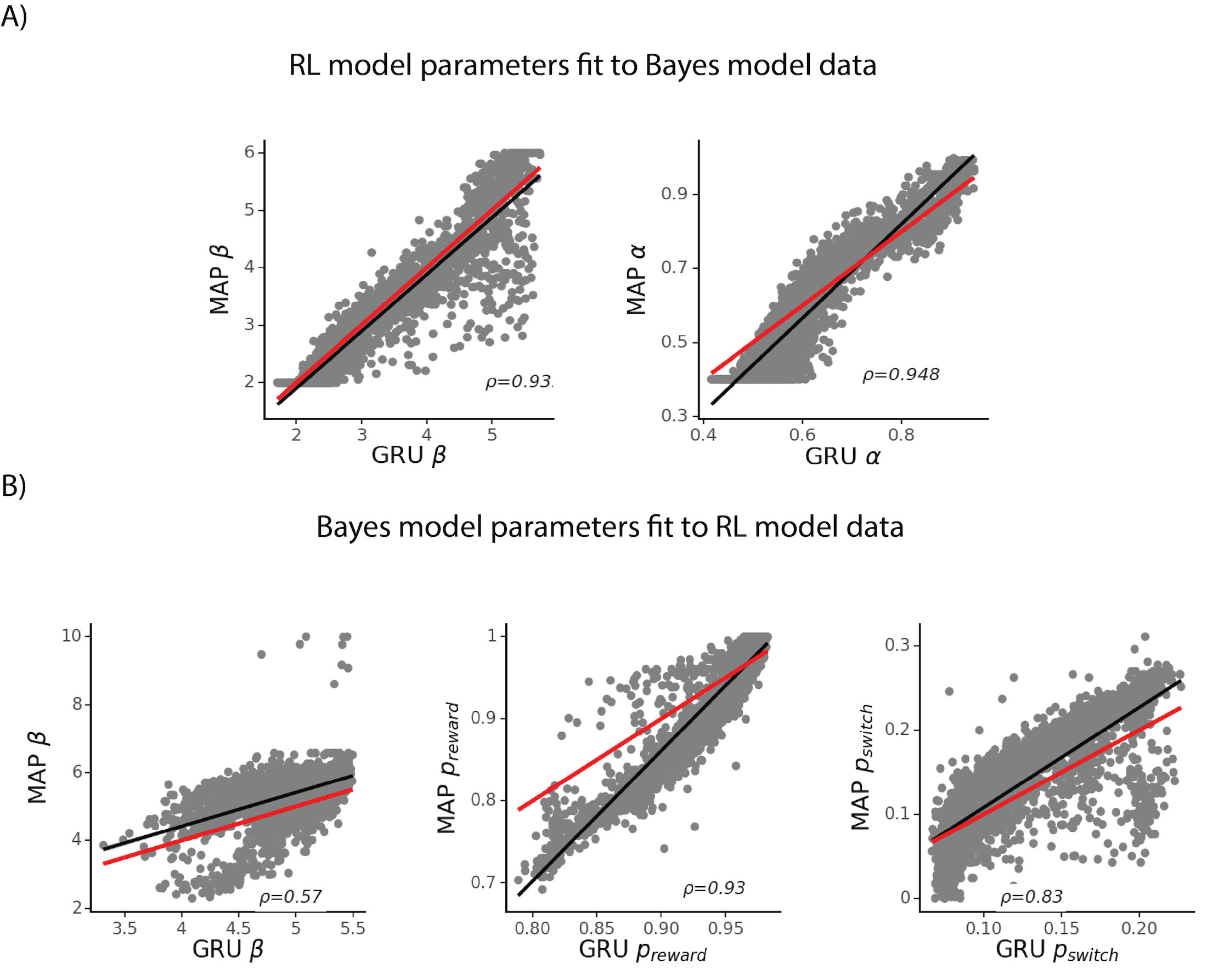

Supplement: S11 Fig — A) The correlation between MAP and GRU RL model parameter estimates, fit to data simulated from Bayesian Inference model. B) The correlation between MAP and GRU Bayes model parameter estimates, fit to data simulated from the RL model. High correlation would imply similarities in estimates between MAP and GRU, suggesting that ANNs are similarly impacted by model misspecification as traditional methods such as MAP. (TIF) [file pcbi.1012119.s011.tif]

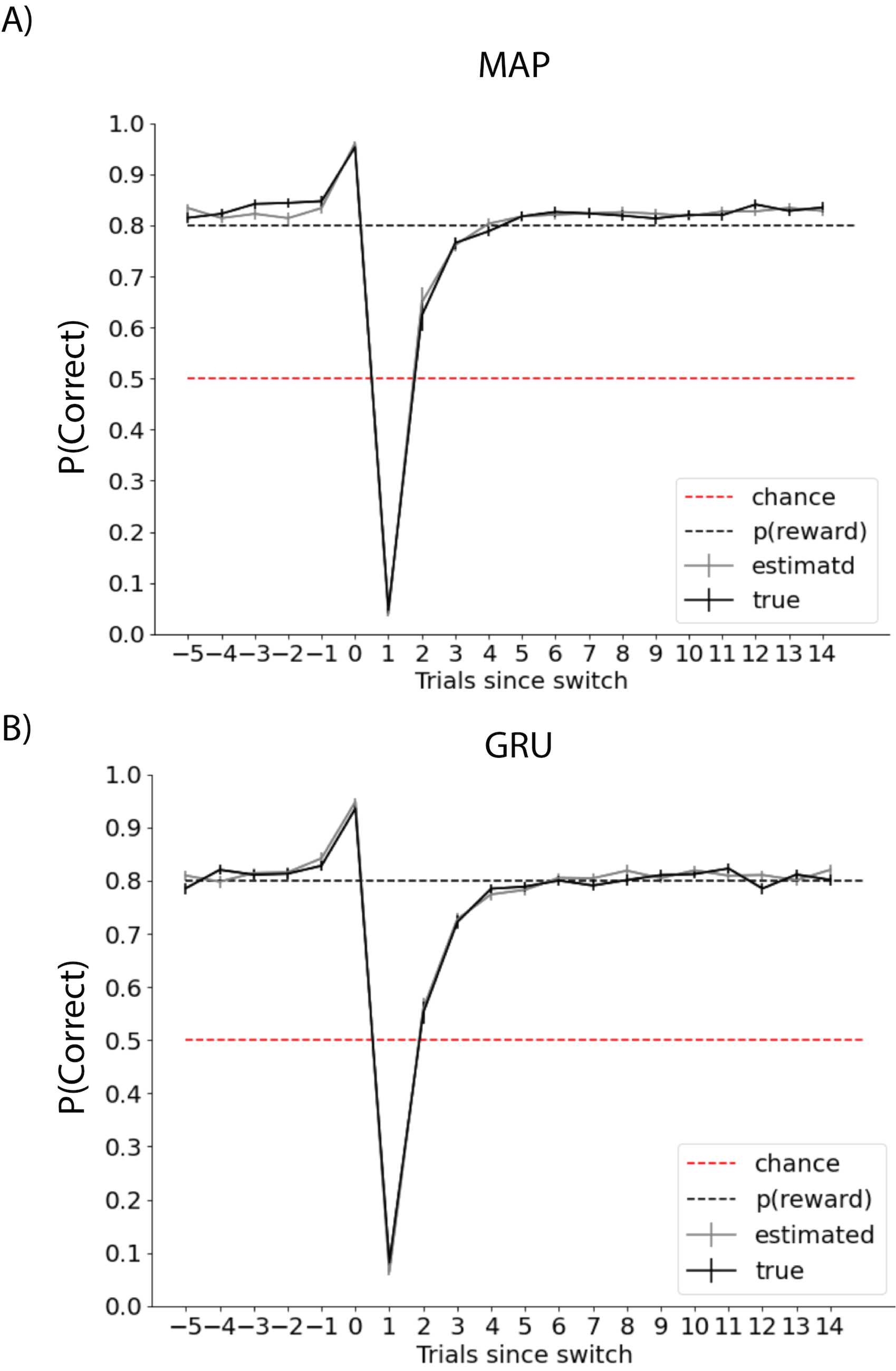

Supplement: S12 Fig — We randomly sampled 100 agents from the test set, and the respective parameter estimates for each of the methods. We simulated data from the model and compared it to ground truth. Both methods successfully recover choices from the ground truth agents. (TIF) [file pcbi.1012119.s012.tif]

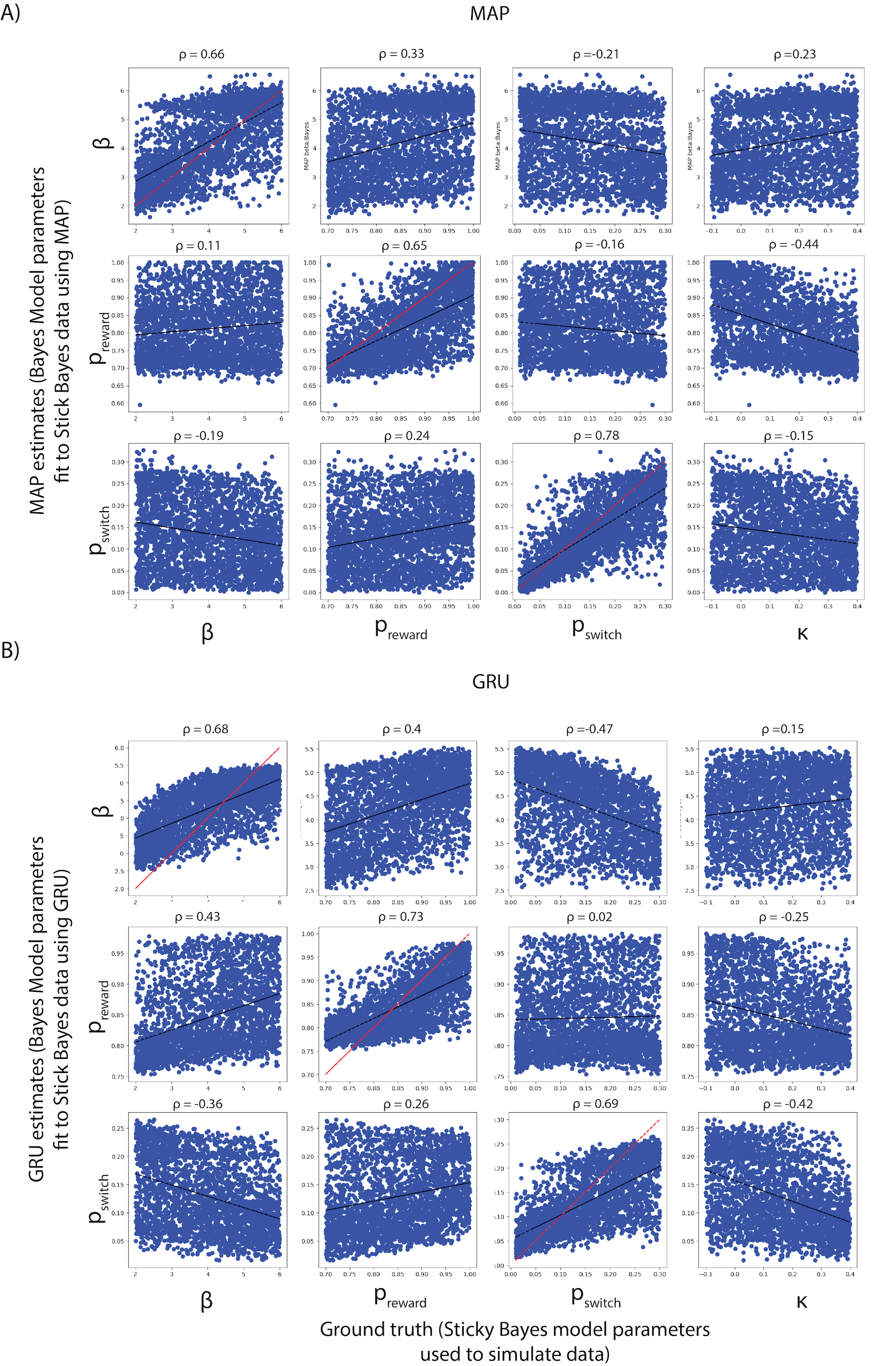

Supplement: S13 Fig — We randomly sampled 100 agents from the test set, and the respective parameter estimates for each of the methods. We then simulated data from the model and compared it to ground truth. Both methods successfully recover choices from the ground truth agents. A) We fit the Bayesian Inference model (without stickiness) to the data simulated from the Bayesian Inference Model with stickiness using MAP. We correlated the estimated Bayesian inference model parameters (y-axis) with the ground truth parameters from the model with stickiness (x-axis). B) For the ANN, we trained the neural network to estimate parameters of the Bayesian inference model, and tested it on the data simulated from the Bayesian inference model with stickiness. We looked at the correlation between the ground truth parameters (from a separate test set), and the predictions of the network trained on the model without stickiness. Both methods show that parameters shared between the misspecified models can be reasonably and similarly recovered. Both ANN and MAP generated some non-zero estimates of stickiness when data simulated from model without stickiness was fit using the model/network that assumes presence of stickiness in the model; however, these values were closely clustered around 0, to a similar degree between methods (S16 Fig). (TIF) [file pcbi.1012119.s013.tif]

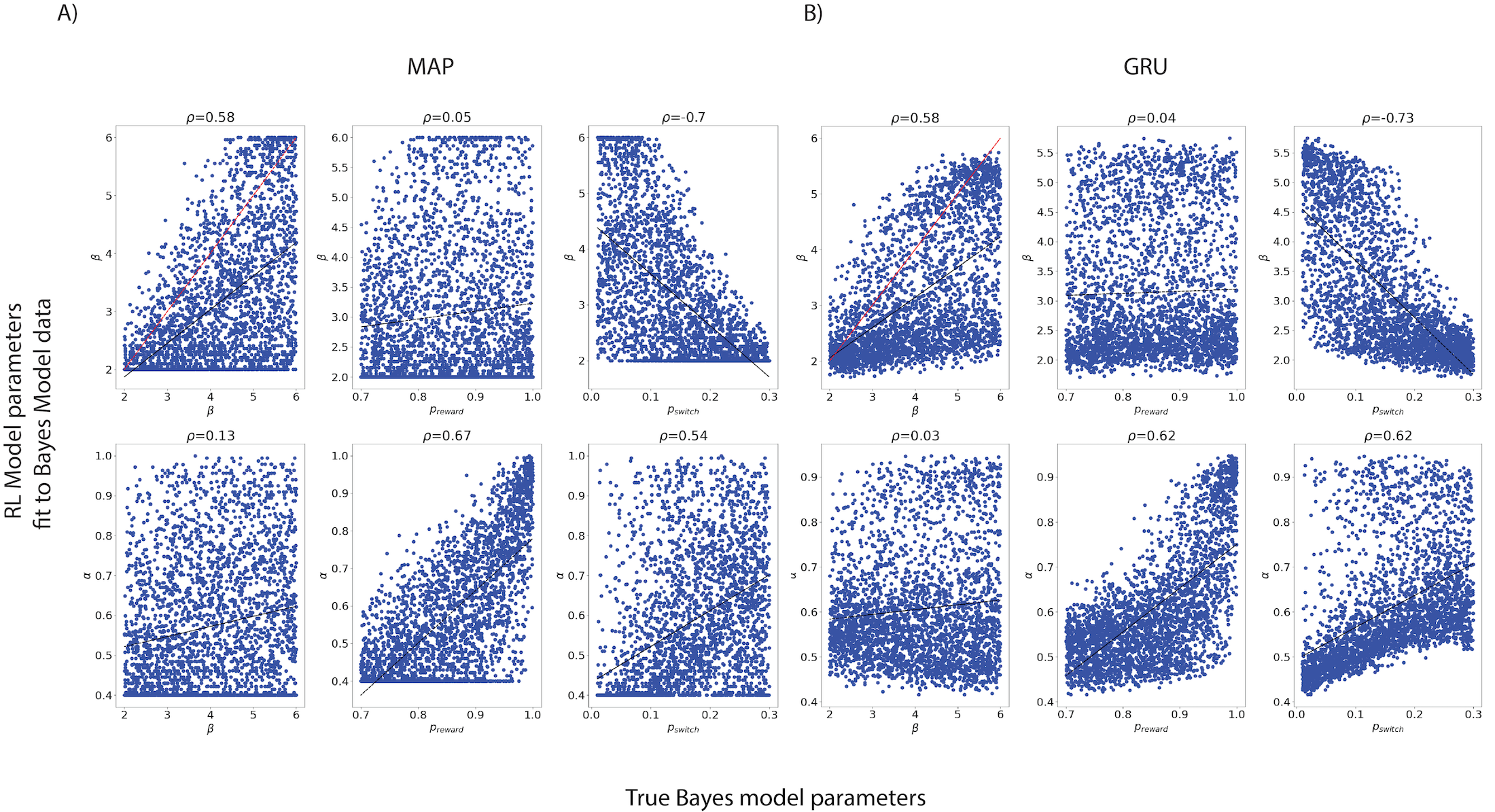

Supplement: S14 Fig — We fit the RL model to the data simulated from the Bayesian Inference model in the probabilistic reversal learning task (see Methods section on Tasks and Cognitive Models) using A) MAP and B) GRU. We correlated the estimated RL model parameters (y-axis) with the ground truth parameters from the Bayesian inference model (x-axis). MAP and GRU show similar patterns between estimated and true parameters, such that variance driven by true parameter β pswitch are both captured in the fit β parameters, while the fit learning rate parameter α captures behavioral variance driven by the Bayesian update parameters preward and pswitch. (TIF) [file pcbi.1012119.s014.tif]

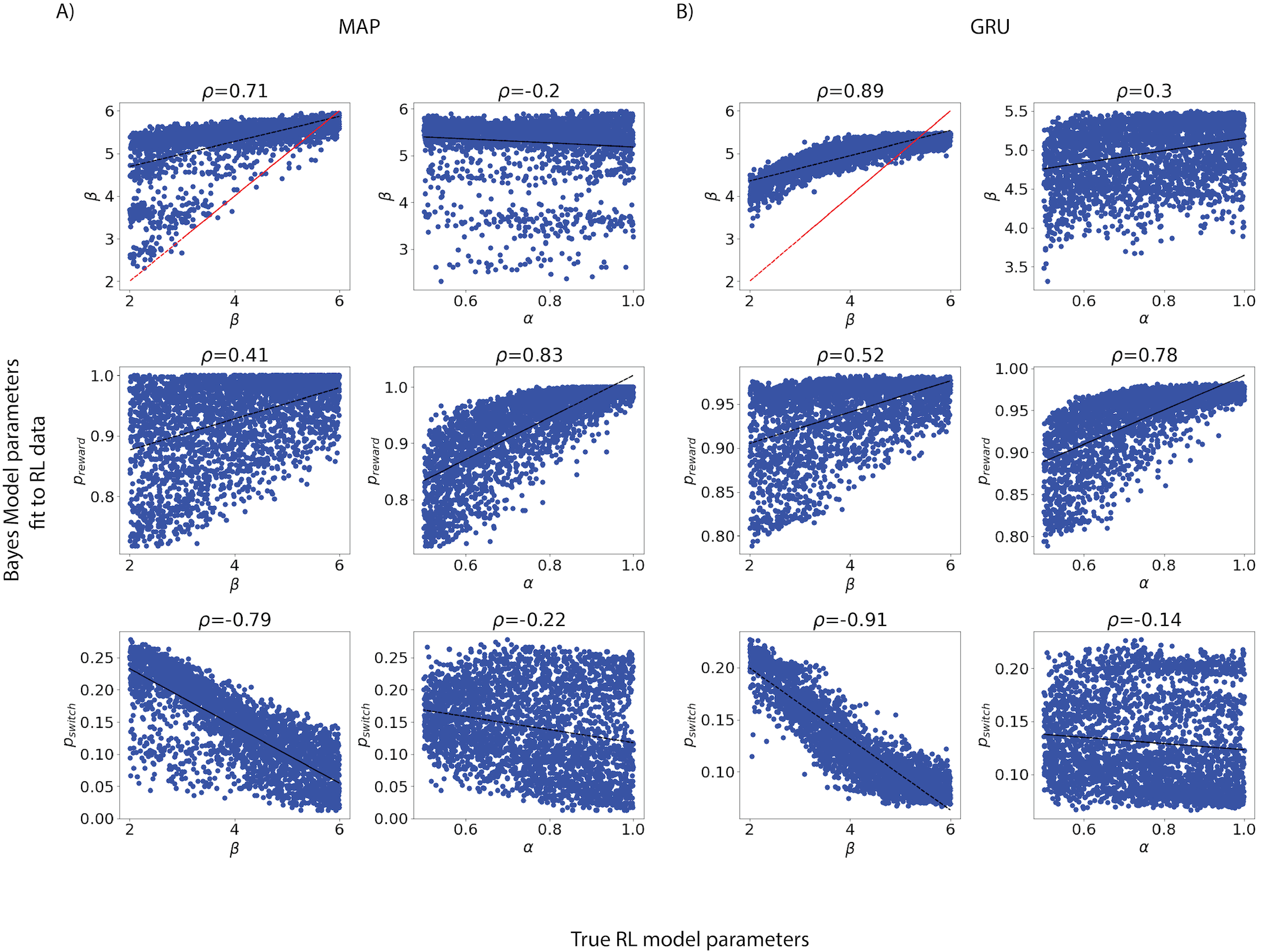

Supplement: S15 Fig — We fit the Bayesian Inference model to the data simulated from the RL model in the probabilistic reversal learning task (see Methods section on Tasks and Cognitive Models) using A) MAP and B) GRU. We correlated the estimated Bayesian inference model parameters (y-axis) with the ground truth parameters from the RL model (x-axis). MAP and GRU again show similar patterns between estimated and true parameters. In particular, we see that in both cases, noise in behavior due to β in the RL model tends to be attributed to the pswitch fit parameter rather than the fit Bayesian model β parameter. Effect of learning rate parameter α are attributed to preward by both methods. (TIF) [file pcbi.1012119.s015.tif]

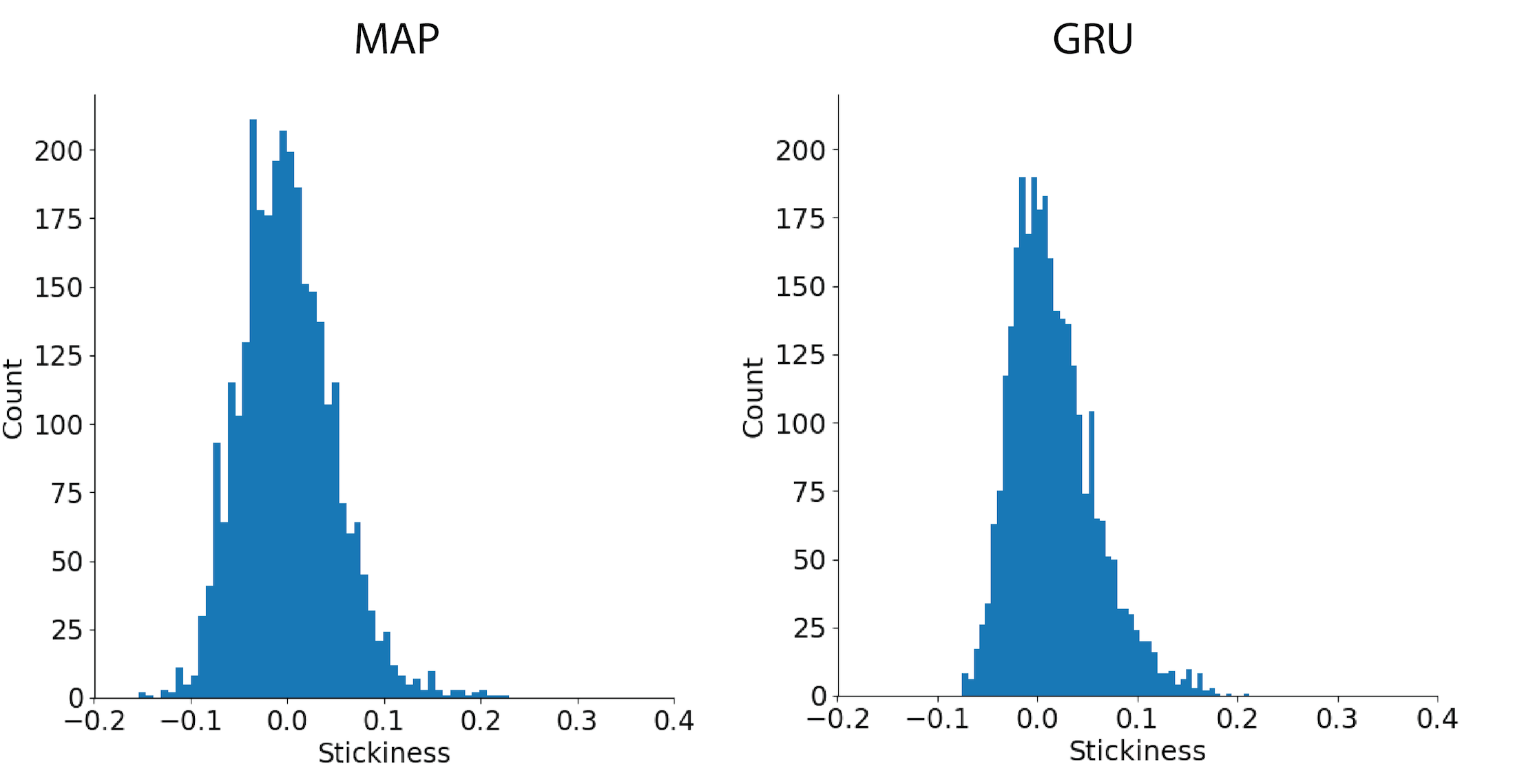

Supplement: S16 Fig — Stickiness parameter estimates from the data simulated from the Bayesian inference model without stickiness from A) fitting the Bayesian inference model with stickiness using MAP, and B) utilizing the ANN trained to estimate parameters of the model with stickiness. Despite both methods producing non-zero estimates of stickiness, they tend to cluster around the value of 0. (TIF) [file pcbi.1012119.s016.tif]

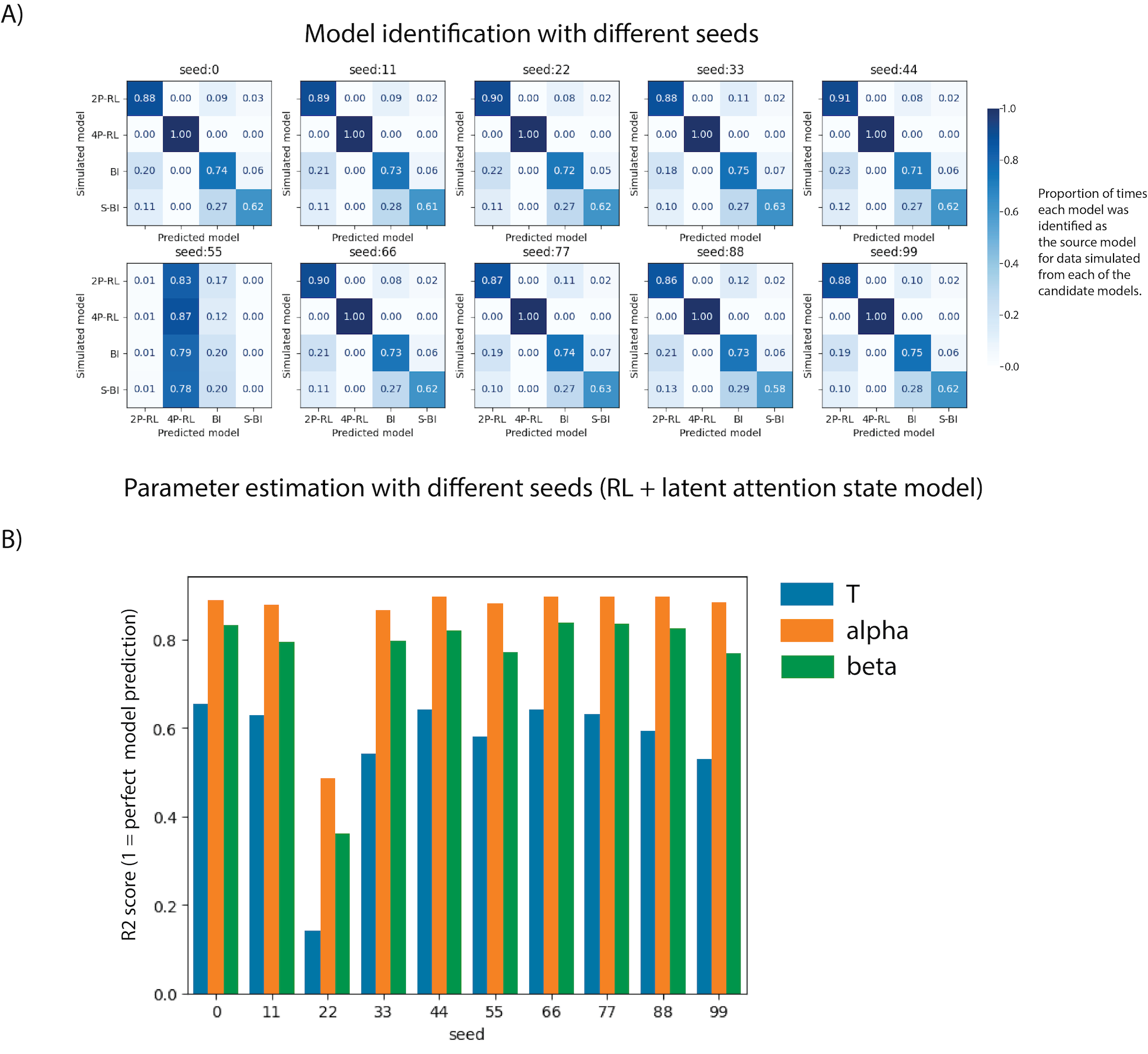

Supplement: S17 Fig — For conciseness, we show tests from 10 different seeds on model identification with 4 models simulated on the PRL task (e.g. same as Fig 5) and parameter estimation of one of the likelihood-intractable models (e.g. RL-LAS model). We found that overall both model identification and parameter estimation had relatively stable results across different seeds, with an exception of one seed value in both cases. (TIF) [file pcbi.1012119.s017.tif]
