# Supplementary material for: Artificial neural networks for model identification and parameter estimation in computational cognitive models
Source: PLoS Comput Biol. 2024 May 15;20(5):e1012119. doi: 10.1371/journal.pcbi.1012119 (PMC11132492; doi:10.1371/journal.pcbi.1012119)
Supplement: Attachment — Submitted filename: Review_PlosComp.pdf [file pcbi.1012119.s019.pdf]

Review of Paper:
Artificial neural networks for model identification and parameter estimation in computational cognitive models

Summary

The paper is part of an emerging field which aims to increase the aspirations of (cognitive) science through the use of modern deep learning methods for model-based inference. Unfortunately, the paper fails to acknowledge nearly all methodological and applied research that happened in the field during the last five years. Considering these developments (see below), none of the currently stated contributions or claims of novelty apply. I believe that the paper needs to be re-written entirely in the sense of applying existing simulation-based methods (also known as amortized Bayesian inference) to interesting cognitive models. Even so, the authors should consider moving away from legacy inference and validation methodology. I hope that some of the related work below can provide a good starting point for getting familiar with current trends, methods, and problems in the field.

Major Points

1 Not Considering Relevant Related Work

The field of amortized Bayesian inference, a subset of simulation-based inference (SBI; Cranmer et al., 2020), has undergone a tremendous revolution in the last five years.

The main idea of amortized (Bayesian) inference consists in a) training a specialized neural network on model simulations and b) applying the trained network on real data. This approach has seen exponential development of methodological innovations, along with multiple applications in cognitive science, neuroscience, computational biology, physics, to name just a few. Below, I provide a non-exhaustive summary (with sometimes overlapping methodological and applied contributions):

- **Parameter estimation with neural networks**: *amortized (i.e., train once, apply to many data sets) posterior methods* (Radev et al., 2023a; Ramesh et al., 2022; Avecilla et al., 2022; Pacchiardi and Dutta, 2022; Schmitt et al., 2021; Radev et al., 2021b, 2020a; Gonçalves et al., 2020; Radev et al., 2019; Ardizzone et al., 2019); *non-amortized (i.e., adaptive inference for a single data set) posterior methods* (Deistler et al., 2022; Durkan et al., 2020; Greenberg et al., 2019; Lueckmann et al., 2017; Papamakarios and Murray, 2016); *neural likelihood (i.e., train a surrogate for the intractable likelihood, combine with MCMC) methods*: (Hermans et al., 2020; Lueckmann et al., 2019; Papamakarios et al., 2019).

  The authors do cite Fengler et al. (2021) Boelts et al. (2022), but I wonder if they ever read the two papers, which do acknowledge some of the references I am including and properly embed their work in the context of simulation-based and amortized inference.

- **Learnable (end-to-end) summary statistics for neural parameter estimation**: (Wrede et al., 2022; Radev et al., 2020a, 2019; Chen et al., 2021; Wiqvist et al., 2019; Chan et al., 2018).
- **Model comparison**: (Schröder and Macke, 2023; Elsemüller et al., 2023; Radev et al., 2023a, 2021a; Pudlo et al., 2016).
- **Applications to cognitive modeling**: (Sokratous et al., 2023; Elsemüller et al., 2023; Schumacher et al., 2022; von Krause et al., 2022; Radev et al., 2020a,b; D'Alessandro et al., 2020; Wieschen et al., 2020; Radev et al., 2019).
- **Widely applicable software for neural SBI**: `TensorFlow` (Radev et al., 2023b), `PyTorch` (Tejero-Cantero et al., 2020). Why rely on custom implementations given the existence of software infrastructure?

  Note, that most of these methods use flexible neural network architectures which are adapted to the probabilistic symmetry of the data (e.g., recurrent networks for time series, deep sets for exchangeable data, etc).

2 Neural Methods Need Proper Diagnostics

The open world setting and model misspecification have been widely acknowledged as problematic for neural SBI (Huang et al., 2023; Ward et al., 2022; Schmitt et al., 2021; Radev et al., 2021a; Hermans et al., 2021; Cannon et al., 2022; Pacchiardi and Dutta, 2022), apart from more general treatments of misspecified models. Thus, any application of neural SBI should come with a validation methodology for both closed- and open-world scenarios. Instead of only comparing point estimates to ground truth values, neural methods should strive to follow principled workflows (see, e.g., Schad et al., 2021, for cognitive modeling), especially since sensitivity analysis, calibration, and predictive checks are now computationally cheap due to amortized inference. See also Dalmasso et al. (2021) and Masserano et al. (2022) for a fully frequentist approach to (neural) SBI with coverage guarantees.

3 Beyond Regression and Point Estimates

Point estimates as well as AIC and BIC have been widely criticized for being too simplistic and for ignoring all sources of uncertainty in analysis (Gelman et al., 2014; Vehtari and Ojanen, 2012). While I do not wish to enforce a Bayesian approach, the disadvantages of using point estimates in complex contexts, such as SBI, have been repeatedly noted (e.g., Cranmer et al., 2020; Radev et al., 2020a). Indeed, using neural networks for non-linear regression in SBI has been around for more than 10 years (Blum et al., 2013)[1], but even the early work of Papamakarios and Murray (2016) moved away from point estimates. Moreover, since the authors' ultimate goal is to scale up inferential techniques to intractable cognitive models, issues such as non-identifiability, mimicry, and model complexity need to be taken into account, regardless of the framework (e.g., Bayesian or frequentist). Point estimates and counting the number of parameters simply don't do it.

[1] Admittedly, Blum and François (2010) used a non-linear heteroskedastic approach which estimates the conditional variance of the parameters, along with the conditional mean.

References

Ardizzone, L., Kruse, J., Wirkert, S., Rahner, D., Pellegrini, E. W., Klessen, R. S., Maier-Hein, L., Rother, C., and Köthe, U. (2019). Analyzing inverse problems with invertible neural networks. In *Intl. Conf. on Learning Representations*.

Avecilla, G., Chuong, J. N., Li, F., Sherlock, G., Gresham, D., and Ram, Y. (2022). Neural networks enable efficient and accurate simulation-based inference of evolutionary parameters from adaptation dynamics. *PLoS Biology*, 20(5):e3001633.

Blum, M. G. and François, O. (2010). Non-linear regression models for approximate bayesian computation. *Statistics and computing*, 20:63–73.

Blum, M. G., Nunes, M. A., Prangle, D., and Sisson, S. A. (2013). A comparative review of dimension reduction methods in approximate bayesian computation. *Statistical Science*, 28(2):189–208.

Boelts, J., Lueckmann, J.-M., Gao, R., and Macke, J. H. (2022). Flexible and efficient simulation-based inference for models of decision-making. *Elife*.

Cannon, P., Ward, D., and Schmon, S. M. (2022). Investigating the Impact of Model Misspecification in Neural Simulation-based Inference. arXiv:2209.01845 [cs, stat].

Chan, J., Perrone, V., Spence, J., Jenkins, P., Mathieson, S., and Song, Y. (2018). A likelihood-free inference framework for population genetic data using exchangeable neural networks. *Advances in neural information processing systems*, 31.

Chen, Y., Zhang, D., Gutmann, M., Courville, A., and Zhu, Z. (2021). Neural approximate sufficient statistics for implicit models. *International Conference on Learning Representations*.

Cranmer, K., Brehmer, J., and Louppe, G. (2020). The frontier of simulation-based inference. *Proceedings of the National Academy of Sciences*.

Dalmasso, N., Masserano, L., Zhao, D., Izbicki, R., and Lee, A. B. (2021). Likelihood-free frequentist inference: Confidence sets with correct conditional coverage. *arXiv preprint arXiv:2107.03920*.

Deistler, M., Goncalves, P. J., and Macke, J. H. (2022). Truncated proposals for scalable and hassle-free simulation-based inference. *Advances in Neural Information Processing Systems*, 35:23135–23149.

Durkan, C., Murray, I., and Papamakarios, G. (2020). On contrastive learning for likelihood-free inference. In *International Conference on Machine Learning*. PMLR.

D'Alessandro, M., Radev, S. T., Voss, A., and Lombardi, L. (2020). A bayesian brain model of adaptive behavior: an application to the wisconsin card sorting task. *PeerJ*, 8:e10316.

Elsemüller, L., Schnuerch, M., Bürkner, P.-C., and Radev, S. T. (2023). A deep learning method for comparing bayesian hierarchical models. *arXiv preprint arXiv:2301.11873*.

Fengler, A., Govindarajan, L. N., Chen, T., and Frank, M. J. (2021). Likelihood approximation networks (lans) for fast inference of simulation models in cognitive neuroscience. *Elife*, 10:e65074.

Gelman, A., Hwang, J., and Vehtari, A. (2014). Understanding predictive information criteria for bayesian models. *Statistics and computing*, 24(6):997–1016.

Gonçalves, P. J., Lueckmann, J.-M., Deistler, M., et al. (2020). Training deep neural density estimators to identify mechanistic models of neural dynamics. *Elife*.

Greenberg, D., Nonnenmacher, M., and Macke, J. (2019). Automatic posterior transformation for likelihood-free inference. In *International Conference on Machine Learning*.

Hermans, J., Begy, V., and Louppe, G. (2020). Likelihood-free mcmc with amortized approximate ratio estimators. In *International Conference on Machine Learning*, pages 4239–4248. PMLR.

Hermans, J., Delaunoy, A., Rozet, F., Wehenkel, A., and Louppe, G. (2021). Averting a crisis in simulation-based inference.

Huang, D., Bharti, A., Souza, A., Acerbi, L., and Kaski, S. (2023). Learning robust statistics for simulation-based inference under model misspecification. *arXiv preprint arXiv:2305.15871*.

Lueckmann, J.-M., Bassetto, G., Karaletsos, T., and Macke, J. H. (2019). Likelihood-free inference with emulator networks. In *Symposium on Advances in Approximate Bayesian Inference*.

Lueckmann, J.-M., Gonçalves, P. J., Bassetto, G., Öcal, K., Nonnenmacher, M., and Macke, J. H. (2017). Flexible statistical inference for mechanistic models of neural dynamics. In *31st NeurIPS Conference Proceedings*.

Masserano, L., Dorigo, T., Izbicki, R., Kuusela, M., and Lee, A. B. (2022). Simulation-based inference with waldo: Perfectly calibrated confidence regions using any prediction or posterior estimation algorithm. *arXiv preprint arXiv:2205.15680*.

Pacchiardi, L. and Dutta, R. (2022). Score matched neural exponential families for likelihood-free inference. *J. Mach. Learn. Res.*, 23:38–1.

Papamakarios, G. and Murray, I. (2016). Fast ε-free inference of simulation models with bayesian conditional density estimation. *Advances in neural information processing systems*, 29.

Papamakarios, G., Sterratt, D., and Murray, I. (2019). Sequential neural likelihood: Fast likelihood-free inference with autoregressive flows.

Pudlo, P., Marin, J.-M., Estoup, A., Cornuet, J.-M., Gautier, M., and Robert, C. P. (2016). Reliable abc model choice via random forests. *Bioinformatics*, 32(6):859–866.

Radev, S. T., D'Alessandro, M., Mertens, U. K., Voss, A., Köthe, U., and Bürkner, P.-C. (2021a). Amortized bayesian model comparison with evidential deep learning. *IEEE Transactions on Neural Networks and Learning Systems*.

Radev, S. T., Graw, F., Chen, S., Mutters, N. T., Eichel, V. M., Bärnighausen, T., and Köthe, U. (2021b). Outbreakflow: Model-based bayesian inference of disease outbreak dynamics with invertible neural networks and its application to the covid-19 pandemics in germany. *PLoS computational biology*.

Radev, S. T., Mertens, U. K., Voss, A., Ardizzone, L., and Köthe, U. (2020a). Bayesflow: Learning complex stochastic models with invertible neural networks. *IEEE transactions on neural networks and learning systems*.

Radev, S. T., Mertens, U. K., Voss, A., and Köthe, U. (2019). Towards end-to-end likelihood-free inference with convolutional neural networks. *British Journal of Mathematical and Statistical Psychology*, 73(1):23–43.

Radev, S. T., Schmitt, M., Pratz, V., Picchini, U., Köthe, U., and Bürkner, P.-C. (2023a). JANA: Jointly amortized neural approximation of complex Bayesian models. In *Proceedings of the 39th Conference on Uncertainty in Artificial Intelligence*, volume 216 of *Proceedings of Machine Learning Research*, pages 1695–1706. PMLR.

Radev, S. T., Schmitt, M., Schumacher, L., Elsemüller, L., Pratz, V., Schälte, Y., Köthe, U., and Bürkner, P.-C. (2023b). BayesFlow: Amortized Bayesian Workflows With Neural Networks. *Journal of Open Source Software*, 8(89):5702.

Radev, S. T., Voss, A., Wieschen, E. M., and Bürkner, P.-C. (2020b). Amortized bayesian inference for models of cognition. *arXiv preprint arXiv:2005.03899*.

Ramesh, P., Lueckmann, J.-M., Boelts, J., Tejero-Cantero, Á., Greenberg, D. S., Gonçalves, P. J., and Macke, J. H. (2022). Gatsbi: Generative adversarial training for simulation-based inference. *arXiv preprint arXiv:2203.06481*.

Schad, D. J., Betancourt, M., and Vasishth, S. (2021). Toward a principled bayesian workflow in cognitive science. *Psychological methods*, 26(1):103.

Schmitt, M., Bürkner, P.-C., Köthe, U., and Radev, S. T. (2021). Detecting model misspecification in amortized bayesian inference with neural networks. *arXiv preprint*.

Schröder, C. and Macke, J. H. (2023). Simultaneous identification of models and parameters of scientific simulators. *arXiv preprint arXiv:2305.15174*.

Schumacher, L., Bürkner, P.-C., Voss, A., Köthe, U., and Radev, S. T. (2022). Neural superstatistics: A bayesian method for estimating dynamic models of cognition. *arXiv preprint arXiv:2211.13165*.

Sokratous, K., Fitch, A. K., and Kvam, P. D. (2023). How to ask twenty questions and win: Machine learning tools for assessing preferences from small samples of willingness-to-pay prices. *Journal of Choice Modelling*, 48:100418.

Tejero-Cantero, A., Boelts, J., Deistler, M., Lueckmann, J.-M., Durkan, C., Gonçalves, P. J., Greenberg, D. S., and Macke, J. H. (2020). Sbi–a toolkit for simulation-based inference. *arXiv preprint arXiv:2007.09114*.

Vehtari, A. and Ojanen, J. (2012). A survey of Bayesian predictive methods for model assessment, selection and comparison. *Statistics Surveys*.

von Krause, M., Radev, S. T., and Voss, A. (2022). Mental speed is high until age 60 as revealed by analysis of over a million participants. *Nature Human Behaviour*, 6(5):700–708.

Ward, D., Cannon, P., Beaumont, M., Fasiolo, M., and Schmon, S. (2022). Robust neural posterior estimation and statistical model criticism. *Advances in Neural Information Processing Systems*, 35:33845–33859.

Wieschen, E. M., Voss, A., and Radev, S. (2020). Jumping to conclusion? a lévy flight model of decision making. *The Quantitative Methods for Psychology*, 16(2):120–132.

Wiqvist, S., Mattei, P.-A., Picchini, U., and Frellsen, J. (2019). Partially exchangeable networks and architectures for learning summary statistics in approximate bayesian computation. In *International Conference on Machine Learning*.

Wrede, F., Eriksson, R., Jiang, R., Petzold, L., Engblom, S., Hellander, A., and Singh, P. (2022). Robust and integrative bayesian neural networks for likelihood-free parameter inference. In *2022 International Joint Conference on Neural Networks (IJCNN)*, pages 1–10. IEEE.
