# Supplementary material for: Artificial neural networks for model identification and parameter estimation in computational cognitive models
Source: PLoS Comput Biol. 2024 May 15;20(5):e1012119. doi: 10.1371/journal.pcbi.1012119 (PMC11132492; doi:10.1371/journal.pcbi.1012119)
Supplement: Attachment — Submitted filename: PCB_DLpaper_review_responses.pdf [file pcbi.1012119.s020.pdf]

Dear Dr. Cai,

Enclosed please find our response to the very helpful suggestions from yourself and the three reviewers. We appreciate the opportunity to submit a revised manuscript, and believe that the resulting paper is greatly improved.

Below we address the specific comments raised by each of the reviewers. We marked our responses, and referenced parts of the revised manuscript in blue text. Finally, we have attached a copy of the revised manuscript.

We would like to note that we identified a minor error in the code of one of the cognitive models we simulated. We have now fixed this issue. While the correction does not impact the overall findings, it does result in slight adjustments to specific numerical values, such as correlations in parameter recovery, across various sections of the manuscript. We apologize for the inconvenience.

We appreciate your consideration.

Sincerely,
Milena Rmus, Ti-Fen Pan, Liyu Xia and Anne Collins

Editor's comments:

Both Reviewers 1 and 2 acknowledged the importance of the work for computational cognitive modeling, while pointing out shortcomings that, if addressed, may significantly strengthen the paper. Please address these suggestions as best as you can. Reviewer 3 pointed out a disconnection of the current manuscript from the literature of amortized Bayesian inference.

This is also reflected in the comments of the other reviewers. I believe the disconnection is not intentional but rather reflects a slow penetration of the new development in this field to psychology/neuroscience. Nonetheless, it is necessary to make a better connection to the literature suggested by the reviewers and explain the contribution more fairly and clearly, especially for the target domain of application.

Reviewer #1:

In this manuscript, the authors suggest a simulation-based inference technique to make inferences about the behavioral model and parameters within such a model to fit (in this case behavioral) data. They do this by training a recurrent neural network (RNN) to ingest choices and report appropriate parameters or the posterior over the model identity. Training is based on simulating behavior using a suitable range of parameters and models, and performing supervised learning. The authors show that this works very well in cases in which likelihoods are available (often outperforming traditional likelihood-based methods) and when they are not (often outperforming an approximate Bayesian computation or ABC method). Some tests of robustness are provided.

The manuscript makes a clear and compelling contribution - and offers a method that, if it can be shown to work on the sort of out-of-sample data that humans and animals generate, could become an important weapon in the modeling armory.

Major comments

The manuscript does not really do justice to the ever-growing collection of simulation based inference methods. Although it cites the Boelts et al paper that uses a neural network to generate a likelihood in cases in which likelihoods do not exist, it does not cite or compare itself to other methods such as Lueckmann, Goncalves et al, NeurIPS (2017) which also learn a direct map from sample data, but in this case to posterior distributions over parameters (and not for behavioral data). This seems like a stronger point of comparison since it is often most useful to know the full posterior.

Thank you for your comment. We have now expanded the discussion of our results to be more embedded in the context of the literature you suggested, as well as the broader literature on amortized Bayesian inference. In addition, we have adjusted the language to make the novelty claims more appropriate in the context of other results.

Similarly, it is not the case that it is always necessary to provide ABC with summary statistics - there are learning methods for this.The review paper on 'Simulation Intelligence' by Lavin et al (2022) has quite an extensive survey.

Thank you for the correction; we have now adjusted the text accordingly on lines 176-185:

Note that in order to obtain our ABC results we had to perform an extensive exploration procedure to select summary statistics - ensuring reasonable ABC results. Indeed, the choice of summary statistics is not trivial and represents an important difficulty of applying basic ABC (Sunnaker et al., 2013), that we can entirely bypass using our new neural network approach. We acknowledge that recent methods have combined deep neural networks with ABC methods to automate (or semi-automate) construction of summary statistics (Y. Chen et al., 2020;

Fearnhead and Prangle, 2012; Jiang et al., 2017; Lavin et al., 2021; Radev, Mertens, et al., 2020; Radev, Voss, et al., 2020). However, we aimed to explore an alternative approach, independent of ABC/approximation methods, and focused on the ABC instantiation that has been most frequently implemented in the field of cognitive science.

If we understand it correctly, the method for model comparison seems pretty unwieldy, since it is trained discriminatively, needing to be completely retrained every time an additional model is considered. An approximate likelihood method would have a big advantage here.

While it is accurate that our method necessitates training when introducing new models, the process doesn't demand an extensive training set. Notably, both the training time and model size are not excessively prohibitive. Below, we present the relevant training time and model size information derived from the model-identification training (reported in Figure 4 of the manuscript). Furthermore, even in a situation that requires training the network with a big number of models, training and using the network is more time-efficient relative to traditional methods used for model comparison (e.g. full confusion matrix based on the cross-fitted AIC scores).

Batch size: 128<br>10k agents 500 trials	Training time	Model size
2P-RL and RL-LAS	19 min	394KB
HRL, BI, S-BI	26 min	337KB

Furthermore, we note that this issue is not unique to our approach. Other model comparison methods (e.g. BayesFlow (Radev et al., 2020); HBI (Piray et al., 2019)) also seem to be subject to retraining with addition of new models.

That MAP estimation performs worse than the RNN in the case that the likelihood is tractable (and the prior is correct; e.g., fig 2) makes one worry that the optimization scheme employed is inefficient.

Thank you for pointing this out. Parameter estimation via likelihood optimization (including MAP) from models applied to the data with the number of trials typically observed in cognitive experiments is frequently noisy (Katahira, 2016). We ensured that we repeated the optimization process a reasonable number of times with different randomly initialized parameter starting points (e.g. at least 20) to minimize the possibility of being stuck in the local minima. Furthermore, below we report the correlation between parameter estimates derived from MAP and from the ANN with GRU units; estimated values of the learning rate parameter (the parameter with the worst recovery) are correlated for the 2 methods, suggesting model parameter identifiability limitations rather than optimization issues.

We understand the concern that MAP is expected to be optimal, and that it is thus unexpected to see the method outperform MAP. However, we think this concern is mitigated by multiple points. First, optimality is under true priors (ours are approximate) and not necessarily with respect to MSE for each parameter; second our method only very slightly overperforms and is trained on a measure directly related to MAP. Based on this, we now clarify that we do not claim our method to be superior (only performing equally well).

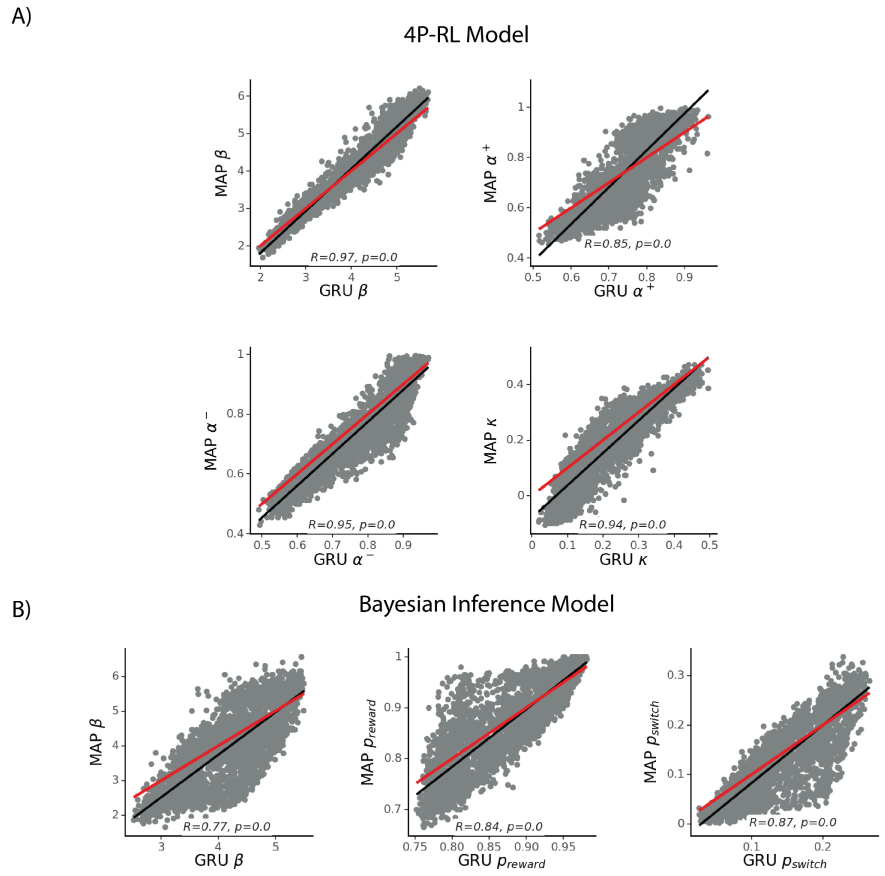

For practical use, it would be important to understand a bit more about things like the impact of the size of the RNN or other parameter choices - particularly since training recurrent nets has its difficulties.

Thank you for pointing this out. We acknowledge hyperparameter selection in network training (including but not limited to the network size) is non-trivial. We recognize that different hyperparameter values can significantly influence a network's performance (e.g. resulting in overfitting or underfitting). We observed that the network performance, particularly in parameter recovery, is most significantly influenced by the number of units in the GRU layer and the chosen dropout rate. A suitable range for the number of GRU units is typically between 90 and 256, covering the needs of most cognitive models. A dropout rate within the range of 0.1 to 0.2

is generally sufficient. We have outlined the details of parameter ranges we tested in the table in supplementary materials.

Furthermore, to address this challenge of hyperparameter selection, we employed bayesian optimization for tuning hyper-parameters that helps reduce the time required to obtain an optimal parameter set by learning from previous iterations. We have now included these details along with the comments on hyperparameter tuning on lines 333-348.

The tests of robustness were a little underwhelming. The effect of partial model mis-specification on parameter estimation (e.g., train with no lapse parameter; fit on data with a lapse parameter) would be interesting.

Thank you for the suggestion, we have now extended the robustness tests. Below we include the results of further analyses testing the effects of model misspecification on both ANN and MAP parameter estimation. A) We fit the Bayesian inference model (without stickiness) to the data simulated from the Bayesian inference model with stickiness using MAP. We correlated the estimated Bayesian inference model parameters with the ground truth parameters from the model with stickiness. B) For the ANN, we trained the neural network to estimate parameters of the Bayesian inference model, and tested it on the test set data simulated from the Bayesian inference model with stickiness. We looked at the correlation between the ground truth Bayesian inference with stickiness model parameters, and the predictions of the network trained on the model without stickiness. We derived the following insights based on this analysis:

First, our results suggest that the parameters shared between the 2 models are reasonably recoverable using both MAP and ANN (e.g. the recovery is noisier but comparable to that of parameters in Bayesian Models without model misspecification; see supplementary figures S4 and S5; furthermore the correlation between ground truth and estimated values is similar for the 2 methods).

We found that for both methods, the stickiness parameter did not trade off with the parameters accounted for by the Bayesian inference model without stickiness.

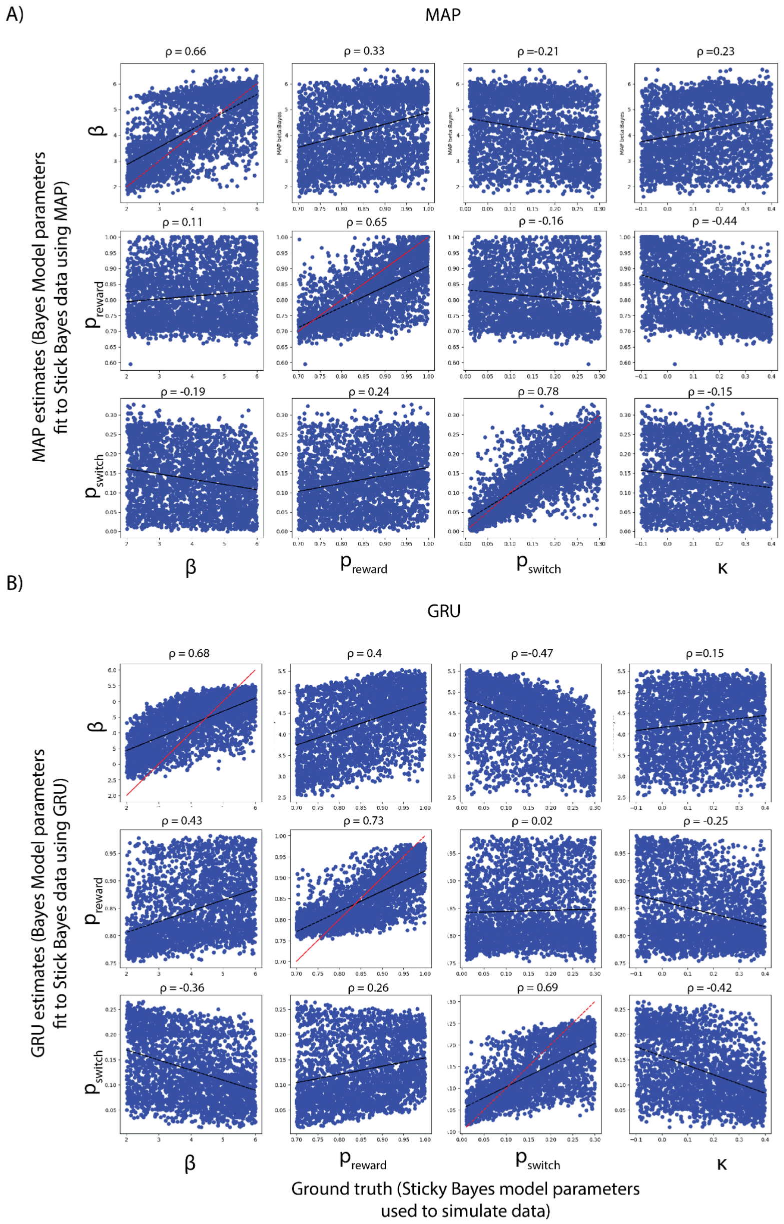

The network trained to estimate parameters from the model with stickiness generated some non-zero stickiness estimates when tested on the data simulated from the model without stickiness (although the majority of the estimates were around zero, and the network was trained on the stickiness range [-0.2 , 0.4]). Nevertheless, the same was true for MAP (e.g. when fitting the data simulated from Bayesian model without stickiness using the Bayesian model with stickiness):

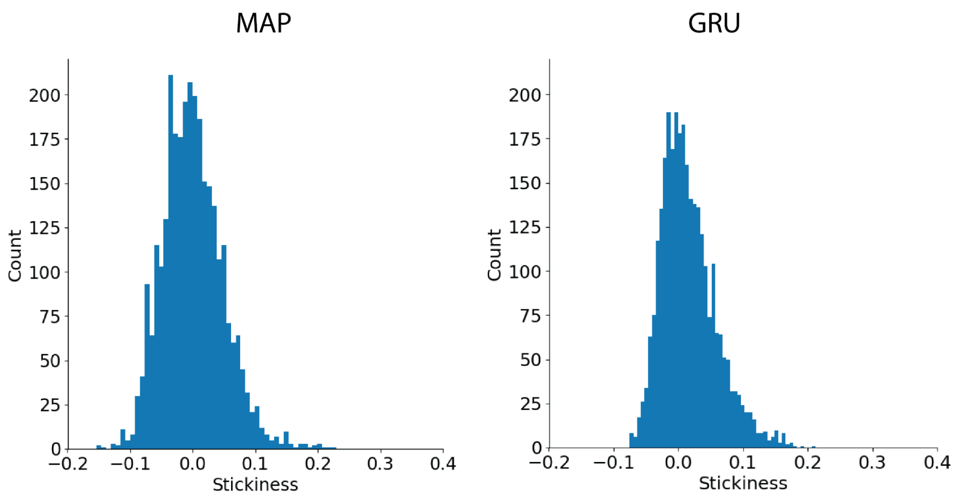

Our main takeaway from this test is that ANN is not any more impacted by model misspecification than the standard methods, especially when it comes to parameters shared between the two models. The fact that the network generates non-zero estimates of stickiness from the data that is generated without stickiness might be explained by the repetitive action sequences that the network attributes to stickiness (given the features it was trained with), which is mostly estimated as close to 0; the same is true for MAP.

We have now added this analysis to the main results (Robustness tests section), which references the figures from the supplementary materials (Supp. Figure S12) .

Uncertainty, as delivered by fully Bayesian approaches, can serve to identify problems with parameters trading-off. It might be worth discussing the possibility of uncertainty estimates through this method.

Thank you for the suggestion. We agree that full uncertainty estimates provide more information compared to point estimate values. However, our current network implementation lacks the capability to offer uncertainty estimates. Future iterations of this tool could potentially address this limitation. Given the potential of evidential deep learning to address epistemic uncertainty, a viable approach involves incorporating learned evidential distributions (Amini et al., 2020) that offer estimates of uncertainty. We've incorporated this consideration into the discussion chapter on lines 380-386.

In Fig 2a, the mistakes the RNN makes for alpha^+ seem quite systematic, could a word be said about why the model cannot overcome these errors?

Thank you for pointing this out, we agree that the errors are somewhat systematic. Below we again show the correlation between parameter estimates via MAP and parameter estimates via ANN (we chose MAP as it was the benchmark method with best parameter recovery). High correlation between MAP and GRU estimates for alpha$^+$ parameter suggests that MAP and

GRU fail in parameter estimation in a similar way. This suggests that the relatively weaker recoverability of the alpha parameter is driven more by weaker identifiability in the model and data than by the specific method limitation. We discuss that while ANNs offer more flexibility in the range of models we can fit to the data, they do not represent a way to fix all the issues that are present in computational modeling on lines 295-306 of the discussion section. We have now included this analysis in the supplementary materials to reference in our discussion paragraph.

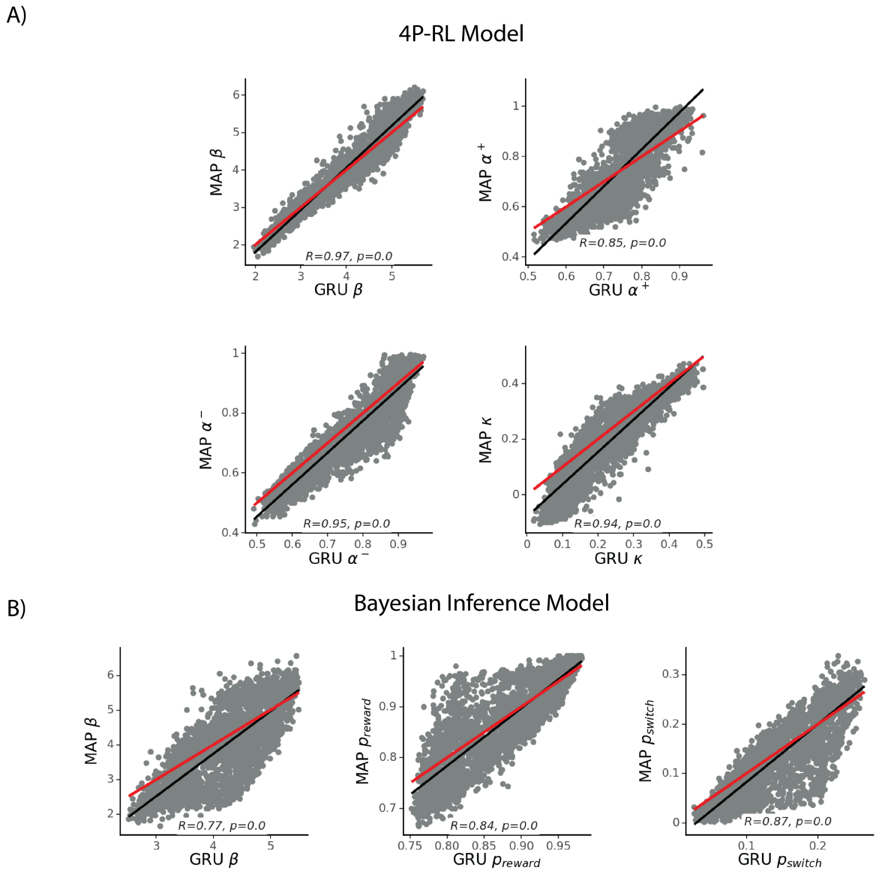

<u>Minor points:</u>

There are some articles missing, e.g. line 112: "across the respective", line 118: "While the fitting process", line 120: "models, the ANN performed" or "models, ANNs performed", and a few more throughout the text.

The unity line is y=x, not x=x.

Thank you for pointing those out, we have carefully edited the text for similar issues.

In line 102 the abbrevations MLE and MAP are given in the wrong order.

Thank you for pointing this out. We have fixed the order.

LLH is used in Figure 1 but never spelled out as log-likelihood.

We have now included the definition of LLH in the figure caption.

Line 191 and elsewhere, we think it should be the number of trials, not length.

We have changed the trial length to trial number or trial sequence length throughout the manuscript.

The caption in Figure 5 mention RL-LAS and 2P-RL, possibly the latter should be HRL?

Thank you for catching that, it is correct it should be HRL; we have fixed the figure caption.

Reviewer #2:

Computational cognitive models of behavior play key roles in psychology and neuroscience. They are at their most powerful when they provide quantitative predictions about behavioral and neural data, which requires fitting their parameters to a dataset. Standard ways of doing this involve computing the likelihood function p(dataset | model, parameters). However, interesting cognitive models exist for which this function is intractable to compute, making them difficult to use. This paper explored a novel and creative solution to this problem: training artificial neural networks on the key tasks of parameter estimation and model comparison. For parameter estimation, this involves generating a large number of synthetic datasets using a single model with known generative parameters, and training an ANN on the mapping between dataset and parameters. For model selection it involves generating synthetic datasets from a variety of different known models, and training an ANN on the mapping from dataset to model identity. Authors show that both methods work well on synthetic datasets.

Overall I think this is a great paper. The problem addressed is an important one. The approach is creative and an interesting new direction for the field. I do think the manuscript could do more to discuss possible limitations which seem likely to arise in moving from synthetic datasets generated by artificial agents to the intended application of laboratory datasets generated by humans or animals. I also think it could do more to discuss pros and cons of the approach relative to a broader range of previously-published alternative approaches.

Major comments

1. A known issue with ANNs is that they can behave incorrectly and unpredictably when given inputs outside the range of their training distribution. In this paper's approach, the training distribution consists of behavior datasets generated by one or more of the cognitive models being considered. In general, it seems to me that we should expect laboratory datasets to be at least somewhat outside of this distribution, even for the very best cognitive models ("all models are wrong"), raising the possibility that ANN-based parameter estimates might be incorrect or

inconsistent. Note that parameter estimates from wrong models can still be scientifically useful, for example in characterizing behavioral differences between experimental groups (and also that even our very best models are almost certainly at least slightly wrong).

Thank you for the comment. We agree that inconsistencies between the training and testing data ranges can result in a reduced network performance (see supplementary figure S9). We also agree that the subjects' data might be noisier than the simulated data, and that it might be expected that parameter ranges based on real subject data might be more widely distributed than the parameter range we specified for the training. Nevertheless, there is a fixed plausible range parameter values are expected to be in (e.g. parameters that are bound between 0 and 1, such as the learning rate). We believe that by using data generated from a full plausible range of parameter values (e.g. by sampling parameters from the full range) one can lessen the concern that the network will underperform due to data being likely generated by the out-of-range parameters. We additionally asserted the network's ability to accurately estimate parameters from shorter test data sequences (representative of noisier data).

We also agree that the accuracy of network parameter estimates does not guarantee that our model is correct, or that a better model doesn't exist - same issues that are present in standard methods (see discussion paragraph lines 291-314); we only propose ANN as a tool that can help us fit parameters from the broader range of computational models, and make no claims that it isn't sensitive to the general limitations of cognitive (or any kind of) models. We now further emphasize this point in the discussion section.

A revised manuscript might explore the extent of this issue further by performing parameter estimates for models other than the generative model (either using laboratory datasets or mis-matched synthetic datasets).

Are these parameter estimates consistent within a dataset between different ANN random seeds? For models with tractable likelihoods, are they consistent with the parameter estimates produced by classic methods? Alternatively, if authors consider this exploration out of scope, a revised manuscript should at least contain a careful discussion of these issues.

Thank you for your suggestion. Please see the results attached below:

1. **Model misspecification (e.g. simulating data from M1, testing the network trained to estimate parameters of M2)**

   Please see the attached figure (fitting data simulated from a Bayesian inference model with stickiness using the network trained on parameter estimation of the Bayesian inference model without stickiness); we found that the ANN is not affected by model misspecification any more than a standard fitting method, as it similarly recovers parameters that are shared between the two models; we also found that the network trained on the bayesian model with stickiness predicts non-zero stickiness estimates from data generated without the stickiness parameter; however these stickiness values are mostly close to 0 (e.g. significantly narrower range compared to the range the

network was trained on that allows for stickiness values that substantially deviate from 0 - [-0.2,0.4]), and the same was true for standard model fitting method (MAP).

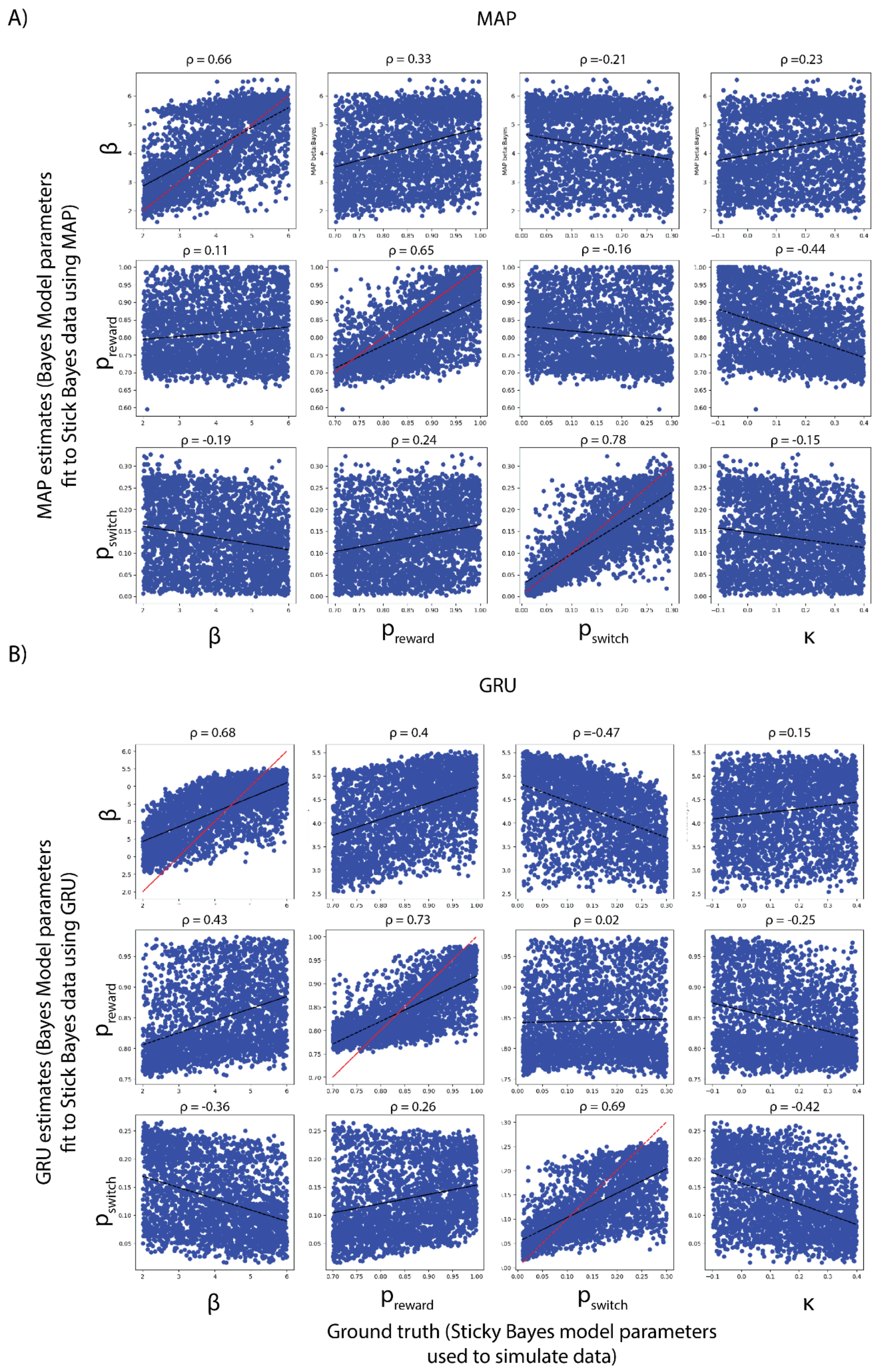

2. Different seed results

A)

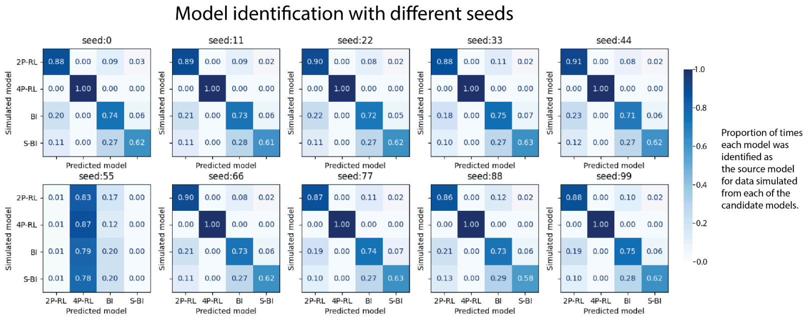

Parameter estimation with different seeds (RL + latent attention state model)

B)

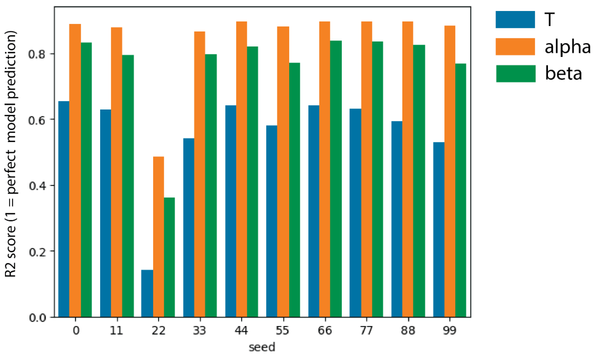

Thanks for the suggestion. In general, we observed small variability in results across different seeds for both parameter estimation and model identification, with only a single seed showing an exception in both cases. These findings have now been incorporated into our supplementary materials. To provide a comprehensive overview, we've expanded our discussion section to highlight the overall stability observed across various seeds, but also underscore the importance for potential users to assess the network performance with different seeds for completeness.

3. Consistency between fitting methods

Please see below a figure showing a correlation between MAP and GRU estimates for the 4P-RL and Bayesian Inference models. Parameter estimation using classic methods is consistent with ANN parameter estimation (for conciseness we have included only parameter estimation from 4P-RL and Bayesian Inference models, but have also checked other models). We now include this in the supplementary material (fig. S10) and reference it in the results section on lines 153-161.

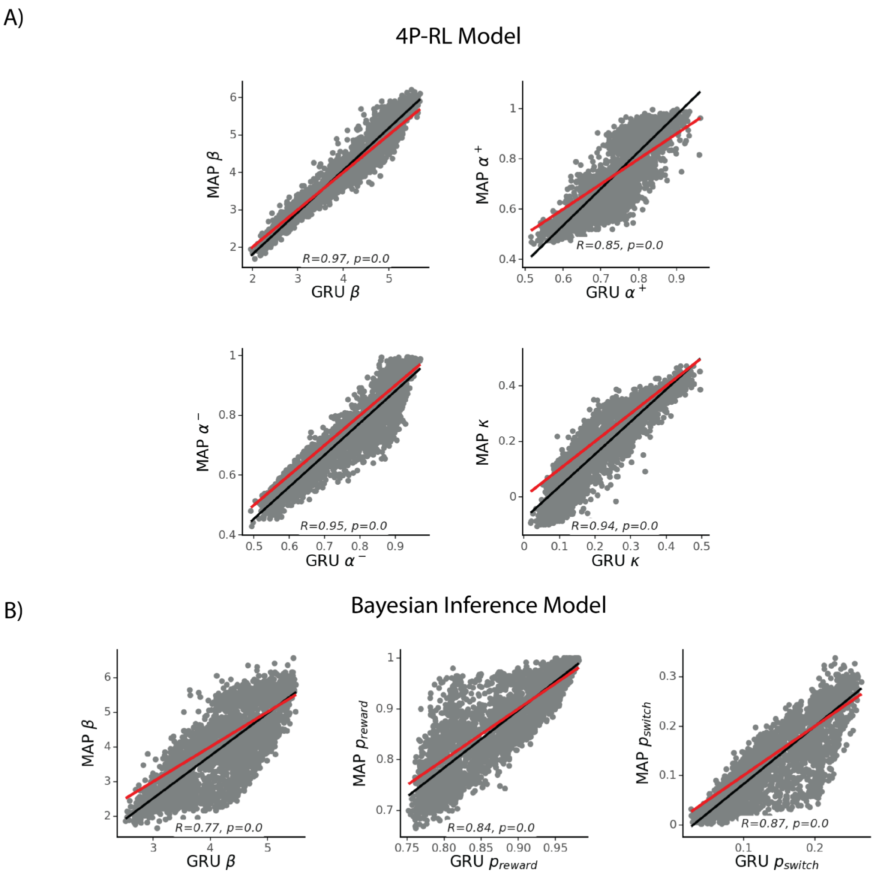

2. The introduction lists a number of alternative approaches to parameter estimation using models with intractable likelihood functions: Approximate Bayesian Computation, Inverse Bayesian Sampling, particle filtering, assumed density estimation, and likelihood approximation networks. Of these, only likelihood approximation networks and ABC are engaged with in detail in the discussion section. It would strengthen the paper to briefly engage with each of the other methods, explaining pros and cons of the ANN-based approach. In particular, the Inverse Bayesian Sampling approach seems to me to have many of the advantages cited for the ANN-based approach: it allows treating the cognitive model as a black box from which behavioral data are sampled, and does not require the user to think in any detail about the model's inner workings. IBS may have advantages over the ANN approach (it likely does not suffer from the training-distribution issue described above), and likely has disadvantages as well (perhaps it is computationally inefficient; certainly its computational demands scale with the number of subjects, while the ANN can perform its expensive computations once then fit new subjects very cheaply; etc). It would be helpful for the manuscript to include the authors' thoughts on this and on other points of comparison between their method and previously-published methods for parameter estimation in models with intractable likelihoods.

Thank you for your suggestion. We have now expanded our comments on alternative methods, and in particular IBS, in the discussion section on lines 360-366:

3. Model selection raises a number of issues, many of which are not unique to the ANN-based approach, but which should be discussed in its context to assist readers. These issues are particularly acute when it comes to selection among nested models. It seems likely to me that the ANN-based approach shares a key limitation with the classic Bayes Factor approach (e.g. via the Savage-Dickey method), in that model selection results will be highly sensitive to the prior over model parameters. This is a major limitation because there is rarely a scientifically principled way to select such a distribution, and because changes in the distribution can easily swing a model comparison from "very strongly favors the simpler model" to "very strongly favors the more complex model". It seems to me that the ANN approach is likely to share this limitation, with the prior over parameters expressed as the generative distribution of parameters used to create the ANN's training dataset. If the authors agree, I think this should be briefly discussed.

Thank you for your comment. We agree that our method is not exempt from the common issues associated with model selection, including that of nested models. We have now pointed this out in the discussion (lines 301-306):

Furthermore, when it comes to model identification, particularly with nested models, the specific parameter ranges can influence the outcome of model identification, favoring simpler models over more complex ones (or vice versa). This was evident in our observations regarding the confusion between Bayesian inference models with and without stickiness, wherein the ground truth values of stickiness played a decisive role in the model identification.

Regardless, it would be helpful to readers to discuss briefly what kinds of scientific conclusions can and cannot safely be drawn from an ANN-driven model selection. Some of these limitations will be shared with classic methods: for example it is always a worry that the "true" model might not be among the set of models being compared – this worry might be particularly acute here if the dataset puts the ANN outside its training distribution.

Thank you for your suggestion. We currently address this concern on lines 291-314 in the discussion section by stating that using ANNs for fitting computational models is not a fault-free approach, merely a more flexible one that still requires hand-crafted cognitive model specification, and that the concerns such as 'do we indeed have the best possible model of behavior' apply to ANNs just as much as they do to the standard methods. We believe that this is a different question altogether, and one that has recently been addressed by a different approach that aims to invert the model fitting process and look for latent patterns in the data by using the ANNs without formally specifying cognitive models, albeit at some degree of loss of specificity granted by the computational cognitive models (Eckstein et al. 2023).

Nevertheless, we have slightly expanded the discussion to emphasize what conclusions can and cannot safely be drawn using our method - namely, similar to other model-fitting approaches, we can mostly draw relative conclusions - with respect to the class of models explicitly tested, and no absolute validation of any specific model.

Minor comments

1. “... for most models, [researchers can] simulate synthetic data to make predictions”. Surely this is the case for all models? In what sense is something a “model” if it can’t be used to simulate data and make predictions?

We agree with the reviewer in theory. In practice, some models may be complex enough that simulations may be costly enough to limit their use as simulators. For example, a complex biologically plausible neural network including ms-resolution ion channel dynamics can make behavioral predictions, but might be sufficiently slow to simulate to limit its practical use in our context (Hazy, Frank, & O’Reilly, 2007; O’Reilly and Frank, 2006.

2. Figure 1: What is “LLH”? I do not think this is defined anywhere

Apologies for missing this. We have now clarified that LLH refers to log-likelihood in the figure caption.

3. Estimating quality of parameter estimation using parameter-by-parameter MSE is a nonstandard choice and should be discussed. It raises the possibility that parameter estimation accuracy will depend critically on model parameterization (for example it will change if parameters are rescaled, or reparameterized to orthogonalize them). It is also likely the explanation for the otherwise-paradoxical finding that ANNs outperform MAP, which is a Bayes optimal way to estimate parameters. For some scientific applications, like comparing parameter estimates between experimental groups, there might be a case to be made that MSE is actually better than standard approaches. For others it is likely worse (though perhaps negligibly so) or equivalent. These things should be discussed for the benefit of the reader.

Thank you for your comment. We used parameter specific MSE as a way to quantify and visualize differences in performance with more granularity - indeed, we see that some parameters are much more recoverable than others, which would be more difficult to observe if we used global instead of parameter-by-parameter MSE.

ANN training inputs were standardized, but to ensure that the MSE was computed on the parameters in the same scale space across the two methods (MAP and ANN) we ensured that we scaled the MAP parameters in a similar way - therefore, the MSE should be comparable between the methods, and across the parameters.

In addition, we have also quantified the results using spearman correlation (which is not sensitive to re-parameterizing), as well as R2 - both of which are in agreement with the MSE metric. We added a sentence to the methods section (network evaluation paragraph) clarifying that our results are robust to the specific choice of quantification.

4. Figure 2A: Unclear if errorbars reflect variability over “participants” (for a fixed random seed), over network seeds (for a fixed dataset), or something else. Variability over seeds is something

that should be clearly shown. If you were to repeat your complete procedure, which I believe includes fitting multiple models in order to select hyperparameters, many times, how much variability in final results would there be? Likewise for Figure 2B. Is this an example network? If so, make that explicit and show something about the variability among networks. And likewise for the remaining figures. Figures 4 and 5 could show standard deviations over seeds.

We apologize for the lack of clarity; the error bars reflect the variability across agents. We have now made this more explicit. As for the result of using different seeds, we show the effect in the figure below, which is now also included in supplementary material. Broadly, results are highly consistent across seeds, but we agree that it is important to check this and we now convey this in the paper (supplementary figure S14, lines 342-348):

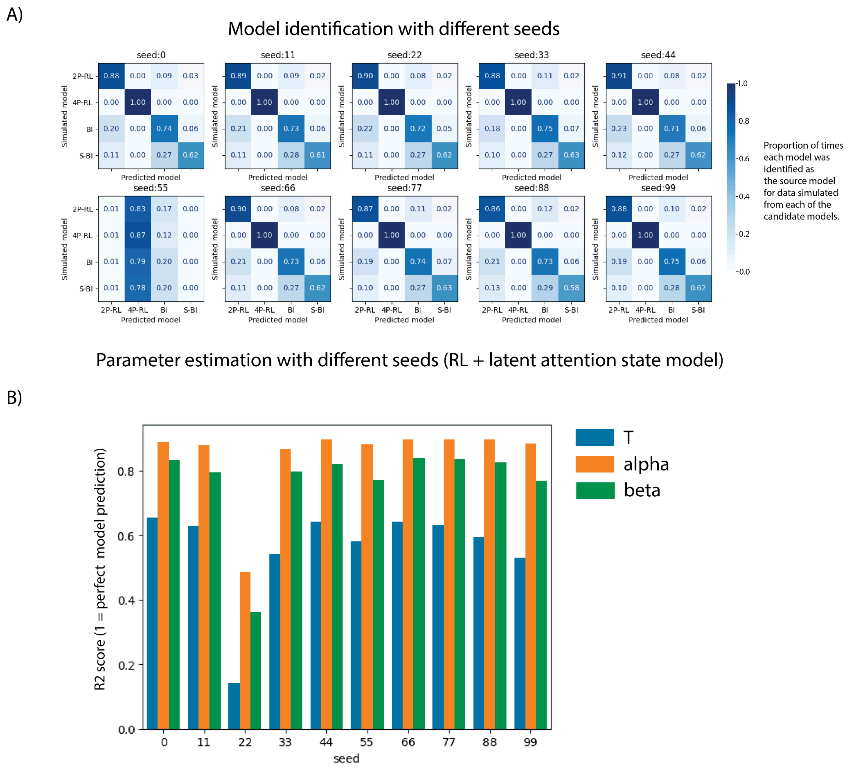

5. “...we cannot compute the likelihood for our likelihood-intractable models” I agree it is not possible to compute this exactly, but my understanding is that techniques do exist for producing unbiased estimates of it (e.g. van Opheusden, Acerbi, and Ma, 2020), and that these could be used to compute an approximate AIC. I don’t necessarily think you need to do this here, but I’d at least tweak the wording to avoid implying that it cannot be done.

We appreciate your suggestion, and we've taken steps to enhance clarity in our language. Specifically, we've clarified that computing the likelihood through closed form solutions via MAP is not feasible. Regarding Inverse Binomial Sampling (van Opheusden et al, 2020), we acknowledge its value; however, it becomes impractical in situations involving models with

sequential dependencies between trials. We have now addressed this aspect in the discussion section.

Reviewer #3:

The paper is part of an emerging field which aims to increase the aspirations of (cognitive) science through the use of modern deep learning methods for model-based inference. Unfortunately, the paper fails to acknowledge nearly all methodological and applied research that happened in the field during the last five years. Considering these developments (see below), none of the currently stated contributions or claims of novelty apply. I believe that the paper needs to be re-written entirely in the sense of applying existing simulation-based methods (also known as amortized Bayesian inference) to interesting cognitive models. Even so, the authors should consider moving away from legacy inference and validation methodology. I hope that some of the related work below can provide a good starting point for getting familiar with current trends, methods, and problems in the field.

Major comments

1. Not Considering Relevant Related Work

The field of amortized Bayesian inference, a subset of simulation-based inference (SBI; Cranmer et al., 2020), has undergone a tremendous revolution in the last five years. The main idea of amortized (Bayesian) inference consists in a) training a specialized neural network on model simulations and b) applying the trained network on real data. This approach has seen exponential development of methodological innovations, along with multiple applications in cognitive science, neuroscience, computational biology, physics, to name just a few. Below, I provide a non-exhaustive summary (with sometimes overlapping methodological and applied contributions):

Parameter estimation with neural networks: amortized (i.e., train once, apply to many data sets) posterior methods (Radev et al., 2023a; Ramesh et al., 2022; Avecilla et al., 2022; Pacchiardi and Dutta, 2022; Schmitt et al., 2021; Radev et al., 2021b, 2020a; Goncalves et al., 2020; Radev et al., 2019; Ardizzone et al., 2019); non-amortized (i.e., adaptive inference for a single data set) posterior methods (Deistler et al., 2022; Durkan et al., 2020; Greenberg et al., 2019; Lueckmann et al., 2017; Papamakarios and Murray, 2016); neural likelihood (i.e., train a surrogate for the intractable likelihood, combine with MCMC) methods : (Hermans et al., 2020; Lueckmann et al., 2019; Papamakarios et al., 2019).

The authors do cite Fengler et al. (2021) Boelts et al. (2022), but I wonder if they ever read the two papers, which do acknowledge some of the references I am including and properly embed

their work in the context of simulation-based and amortized inference.

Thank you for your suggestions and for referencing relevant research. We recognize the significance of this work and admit that, regrettably, we were not previously aware of it despite literature review, discussions with colleagues, and insights from reviewers supporting the grant which funds this work. This unfortunate oversight may be partly attributed to the barriers between different fields. The application of amortized inference to cognitive modeling specifically is a bit of a niche area, and the accessibility of this research might be limited, contributing to our oversight. We have now referenced the relevant work throughout the paper, removed the erroneous novelty claims where relevant, and commented on the ways our approach differs.

2. Neural Methods Need Proper Diagnostics

The open world setting and model misspecification have been widely acknowledged as problematic for neural SBI (Huang et al., 2023; Ward et al., 2022; Schmitt et al., 2021; Radev et al., 2021a; Hermans et al., 2021; Cannon et al., 2022; Pacchiardi and Dutta, 2022), apart from more general treatments of misspecified models. Thus, any application of neural SBI should come with a validation methodology for both closed- and open-world scenarios. Instead of only comparing point estimates to ground truth values, neural methods should strive to follow principled workflows (see, e.g., Schad et al., 2021, for cognitive modeling), especially since sensitivity analysis, calibration, and predictive checks are now computationally cheap due to amortized inference. See also Dalmasso et al. (2021) and Masserano et al. (2022) for a fully frequentist approach to (neural) SBI with coverage guarantees.

Thank you for your suggestions. We have expanded the supplementary materials to include predictive checks, model and parameter range misspecification in addition to the correlations between true and estimated parameters (Supplementary figures S11,S12,S13).

3. Beyond Regression and Point Estimates

Point estimates as well as AIC and BIC have been widely criticized for being too simplistic and for ignoring all sources of uncertainty in analysis (Gelman et al., 2014; Vehtari and Ojanen, 2012). While I do not wish to enforce a Bayesian approach, the disadvantages of using point estimates in complex contexts, such as SBI, have been repeatedly noted (e.g., Cranmer et al., 2020; Radev et al., 2020a). Indeed, using neural networks for non-linear regression in SBI has been around for more than 10 years (Blum et al., 2013), but even the early work of Papamakarios and Murray (2016) moved away from point estimates. Moreover, since the authors' ultimate goal is to scale up inferential techniques to intractable cognitive models, issues such as non-identifiability, mimicry, and model complexity need to be taken into account, regardless of the framework (e.g., Bayesian or frequentist). Point estimates and counting the number of parameters simply don't do it.

Thanks for the suggestion. As stated in response to R1, we agree that uncertainty over parameter estimates provides more information compared to only point estimate values. However, our current network implementation lacks the capability to offer uncertainty estimates. Future iterations of this tool could potentially address this limitation. Implementing a Bayesian neural network is one possibility, as it would supply the uncertainty estimates and could prove to be a valuable architecture to explore; evidential deep learning could be another option.
