# Supplementary material for: Artificial neural networks for model identification and parameter estimation in computational cognitive models
Source: PLoS Comput Biol. 2024 May 15;20(5):e1012119. doi: 10.1371/journal.pcbi.1012119 (PMC11132492; doi:10.1371/journal.pcbi.1012119)
Supplement: Attachment — Submitted filename: Reviewer3_PlosComp.pdf [file pcbi.1012119.s021.pdf]

Second Review of Paper: *Artificial neural networks for model identification and parameter estimation in computational cognitive models*

Summary

I appreciate the authors' effort in revising key notions and claims in the paper as well as acknowledging a subset of the previous work in simulation-based inference (of which the authors' method is a part). I also appreciate the added robustness checks which raise the awareness for the real problems of model misspecification and out-of-distribution data that neural approximators face. Still, I believe there are some crucial misunderstandings regarding the relationship between the current approach and the vast sea of simulation-based inference methods available for solving the tasks at hand.

Major Points

The Integration of ABC and Neural Networks

Most recent neural approaches to simulation-based inference are not *integrated or combined* with ABC, as suggested, for example, by the new paragraph P3L95-100. Rather, they have been **explicitly proposed and developed as an efficient alternative to ABC** (e.g., Sokratous et al., 2023; Ghaderi-Kangavari et al., 2023; Cranmer et al., 2020; Radev et al., 2020; Greenberg et al., 2019; Gonçalves et al., 2020) and this is independent of the particular neural architecture involved, be it a standard regression network, a normalizing flow (e.g., realized as an invertible network), or a diffusion model. The same goes for amortized model comparison (e.g., Radev et al., 2021). The authors' approach is a special case of (amortized) simulation-based inference with learned summary statistics (see below) and point estimates (instead of full posteriors or mean-variance estimates). Moreover, the recent works of Sokratous et al. (2023) and Ghaderi-Kangavari et al. (2023) should be clearly referenced as applications of neural networks for parameter estimation of cognitive model families.

The Role of Summary Statistics

The authors suggest repeatedly that their approach focuses on transforming data to parameters without the selection of summary statistics. This is misleading, as the summary statistics in the authors' lightweight regression approach are merely *implicit*. Consider, for instance, an arbitrary many-to-one recurrent neural network f trained to map sequences $x_{1:T} := x_1, x_2, \ldots, x_T$ to model parameters θ, $\hat{\theta} = f(x_{1:T})$. Clearly, the transformation realized by the network can always be simply decomposed into a learnable compression h (the many-to-one part) followed by a learnable projection

g (the regression part) operation,

$$f(x_{1:T}) = g \circ h(x_{1:T}). \tag{1}$$

Thus, the projection cannot possibly be trained to generate the correct parameters estimates (e.g., the posterior mean in case of using the $L2$ loss or the MAP using standard weight regularization), unless the compression is a sufficient summary statistic for the conditional point quantity of interest. Sticking with the $L2$ loss and the posterior mean, the following must hold for correct point estimation,

$$\mathbb{E}\left[\theta \mid x_{1:T}\right] = \mathbb{E}\left[\theta \mid g(x_{1:T})\right]. \tag{2}$$

In other words, the fact that summary statistics are not considered does not mean that they are not there. Moreover, explicit (fixed or learned) summary statistics are a key proxy for detecting and rectifying the impact of model misspecification (e.g., Ghaderi-Kangavari et al., 2023; Frazier et al., 2020; Frazier and Drovandi, 2021; Schmitt et al., 2021; Huang et al., 2023).

Uncertainty Quantification

The authors can easily extend their parameter estimation approach to a heteroskedastic regression which at least outputs the posterior variance along the posterior mean of the parameters (e.g., Kendall and Gal, 2017; Radev et al., 2019). The extension consists in minimizing the following loss

$$\mathcal{L} = \frac{1}{N} \sum_{i=1}^{N} \left[\frac{\log(\boldsymbol{\sigma}_i^2)}{2} + \frac{(\boldsymbol{\theta}_i - \boldsymbol{\mu}_i)^2}{2\boldsymbol{\sigma}_i^2} \right] \tag{3}$$

in expectation over the simulator outputs, where $\boldsymbol{\mu}$ and $\boldsymbol{\sigma}$ are the outputs of the regression network. I see no technical barriers for enabling this straightforward extension, given the authors' expertise in implementing the current workflows.

References

Cranmer, K., Brehmer, J., and Louppe, G. (2020). The frontier of simulation-based inference. *Proceedings of the National Academy of Sciences.*

Frazier, D. T. and Drovandi, C. (2021). Robust approximate bayesian inference with synthetic likelihood. *Journal of Computational and Graphical Statistics*, 30(4):958–976.

Frazier, D. T., Robert, C. P., and Rousseau, J. (2020). Model misspecification in approximate bayesian computation: consequences and diagnostics. *Journal of the Royal Statistical Society Series B: Statistical Methodology*, 82(2):421–444.

Ghaderi-Kangavari, A., Rad, J. A., and Nunez, M. D. (2023). A general integrative neurocognitive modeling framework to jointly describe eeg and decision-making on single trials. *Computational Brain & Behavior*, pages 1–60.

Gonçalves, P. J., Lueckmann, J.-M., Deistler, M., Nonnenmacher, M., Öcal, K., Bassetto, G., Chintaluri, C., Podlaski, W. F., Haddad, S. A., Vogels, T. P., et al. (2020). Training deep neural density estimators to identify mechanistic models of neural dynamics. *Elife*, 9:e56261.

Greenberg, D., Nonnenmacher, M., and Macke, J. (2019). Automatic posterior transformation for likelihood-free inference. In *International Conference on Machine Learning.*

Huang, D., Bharti, A., Souza, A., Acerbi, L., and Kaski, S. (2023). Learning robust statistics for simulation-based inference under model misspecification. *arXiv preprint arXiv:2305.15871*.

Kendall, A. and Gal, Y. (2017). What uncertainties do we need in bayesian deep learning for computer vision? *Advances in neural information processing systems*, 30.

Radev, S. T., D'Alessandro, M., Mertens, U. K., Voss, A., Köthe, U., and Bürkner, P.-C. (2021). Amortized bayesian model comparison with evidential deep learning. *IEEE Transactions on Neural Networks and Learning Systems*.

Radev, S. T., Mertens, U. K., Voss, A., Ardizzone, L., and Köthe, U. (2020). Bayesflow: Learning complex stochastic models with invertible neural networks. *IEEE transactions on neural networks and learning systems*.

Radev, S. T., Mertens, U. K., Voss, A., and Köthe, U. (2019). Towards end-to-end likelihood-free inference with convolutional neural networks. *British Journal of Mathematical and Statistical Psychology*, 73(1):23–43.

Schmitt, M., Bürkner, P.-C., Köthe, U., and Radev, S. T. (2021). Detecting model misspecification in amortized bayesian inference with neural networks. *arXiv preprint*.

Sokratous, K., Fitch, A. K., and Kvam, P. D. (2023). How to ask twenty questions and win: Machine learning tools for assessing preferences from small samples of willingness-to-pay prices. *Journal of Choice Modelling*, 48:100418.
