# Supplementary material for: Artificial neural networks for model identification and parameter estimation in computational cognitive models
Source: PLoS Comput Biol. 2024 May 15;20(5):e1012119. doi: 10.1371/journal.pcbi.1012119 (PMC11132492; doi:10.1371/journal.pcbi.1012119)
Supplement: Attachment — Submitted filename: PCB_RmusEtAl_Revision2_Letter.pdf [file pcbi.1012119.s022.pdf]

Dear Dr. Cai,

Enclosed please find our response to the suggestions from yourself and the three reviewers. We appreciate the opportunity to submit a revised manuscript.

Below we address the specific comments raised by each of the reviewers. We marked our responses, and referenced parts of the revised manuscript in blue text. Finally, we have attached a copy of the revised manuscript.

We appreciate your consideration.

Sincerely,
Milena Rmus, Ti-Fen Pan, Liyu Xia and Anne Collins

Editor:

Both Reviewers 1 and 3 expressed concerns with not performing an evaluation or implementation of posterior estimation rather than just point estimate. Therefore, it seems necessary to include this demonstration in the paper. Other comments from Reviewers 3 are also important and should be properly addressed. In addition, although not all of the researchers in cognitive modeling are aware of the recent progress in simulation-based inference, given what reviewers have pointed out, and some list of the existing works: https://github.com/smsharma/awesome-neural-sbi, it would be great to not over-state the contribution of the work and provide proper acknowledge of the existing contribution. Demonstrating the method on real data as suggested by Reviewer 2 especially from the domain in which the authors are familiar with would be a good idea to further strengthen the paper.

Thank you for the opportunity to further revise our paper. We have now adjusted the manuscript to include all reviewers' further points, including:

- Uncertainty estimation (as requested by reviewers 1 and 3)
- Further model misspecification analysis (as requested by reviewer 2)
- Clarifying the framing around our vs. reviewer 3's methods, as requested by reviewer 3.

We note that reviewer 2 mentioned "real data" as a way to motivate further model misspecification analysis, rather than requesting we apply the method to real data as summarized by the editor. We have thus followed the reviewer's request and done the further model misspecification analysis.

We hope you will find the paper adequately strengthened, as we do.

Reviewer #1:

The authors have responded well to many of the points of the reviewers. Although the paper does now refer more comprehensively to the wider simulation-based inference literature, it's a little underwhelming not to have implemented/tested alternatives from this stable as a clear alternative (giving extra features such as posteriors rather than point estimates). Nevertheless, the paper's worthwhile contribution is now appropriately hedged.

Thank you for your suggestion. We have added a section called "Uncertainty of Parameter Estimates" (lines 201-217) which presents results with the uncertainty estimates derived from incorporating evidential learning (as outlined by Amini et al.,2020) into our method. Our results show that our approach can be easily adapted to incorporate uncertainty estimation (see lines 694-706 for methods details), without loss to the point estimate (which is typically the goal for our target application), but with a somewhat higher computational cost.

Minor points:
L275: "promise [in successfully arbitrating between competing cognitive models] [in model identification]" The content of only one pair of brackets was probably meant to be in that sentence.

Thank you for catching this. We adjusted the sentence.

L278 "any standard cognitive data set with [a] normal number of participants "

We added the "a".
Figure 3B panel ABC, True T: put "rho=.5" annotation lower

We have adjusted the figure.

Fig S3: The top right plot changed quite a bit (and is now terrible), maybe the authors just want to double-check this is correct?

We have double-checked this, and are confident that this is correct. The change was due to a previous minor bug which artificially enhanced the results for this method.

Fig S6: the p-value should be spelled out as something like "p<1e-xx"

We have adjusted the p-value in the figure.

Reviewer #2:

Thank you for your thorough response to my and the other reviewers' comments. The response fully addresses all of my concerns other than major comment #1, which it seems to

misunderstand. I’ve tried to rephrase this comment below to unpack it a bit more. I’ve also described an analysis which represents one simple way of addressing it which I believe would be very straightforward to do. If the authors prefer some other way, including merely acknowledging the concern in the text, that’s of course fine too.

Thank you for your positive comments and constructive explanation of your major comment #1. We considered that the previous model misspecification analysis we performed was meant to address it, but we now understand that you refer to a stronger form of model misspecification where the models are from a different family altogether. We hope we have now addressed your concern.

Major Comment
One major claim of this manuscript is that ANN-based parameter estimation is a reasonable substitute for MAP-based parameter estimation, and that this is a practical tool that might be useful for fitting models to real datasets generated by humans or animals. The manuscript does a great job of showing that this is true when estimating parameters from synthetic datasets where the generative process exactly matches the model being fit. But real data are never generated from exactly the model being fit (they’re generated by a human or an animal), so it’s important to check whether the ANN-based method returns similar results to MAP when applied to datasets generated by different models.
For concreteness: one way to address this would be creating a plot like the new Figure S10, which compares parameter estimates produced by MAP and by the ANN, but using mis-specified models. For example panel A showing estimates of RL model parameters would analyze datasets produced by the Bayesian model, and panel B vice-versa. Or of course the revision might choose to address it in some other way.

Thank you for your suggestion. We have conducted the model misspecification analysis you suggested, and summarize the results below (these are now included as supplementary figures S11, S14, S15).

We simulated data from the Bayesian inference model, and then estimated RL parameters based on the data sequences by 1) fitting the RL model using MAP and 2) using the neural network trained to estimate RL model parameters. Here we show the correlation between true and estimated parameters for both MAP and GRU methods. The main takeaway from this misspecification analysis is that we observe extremely similar patterns of variance attribution across both methods, such that the impact of model misspecification is comparable across methods.

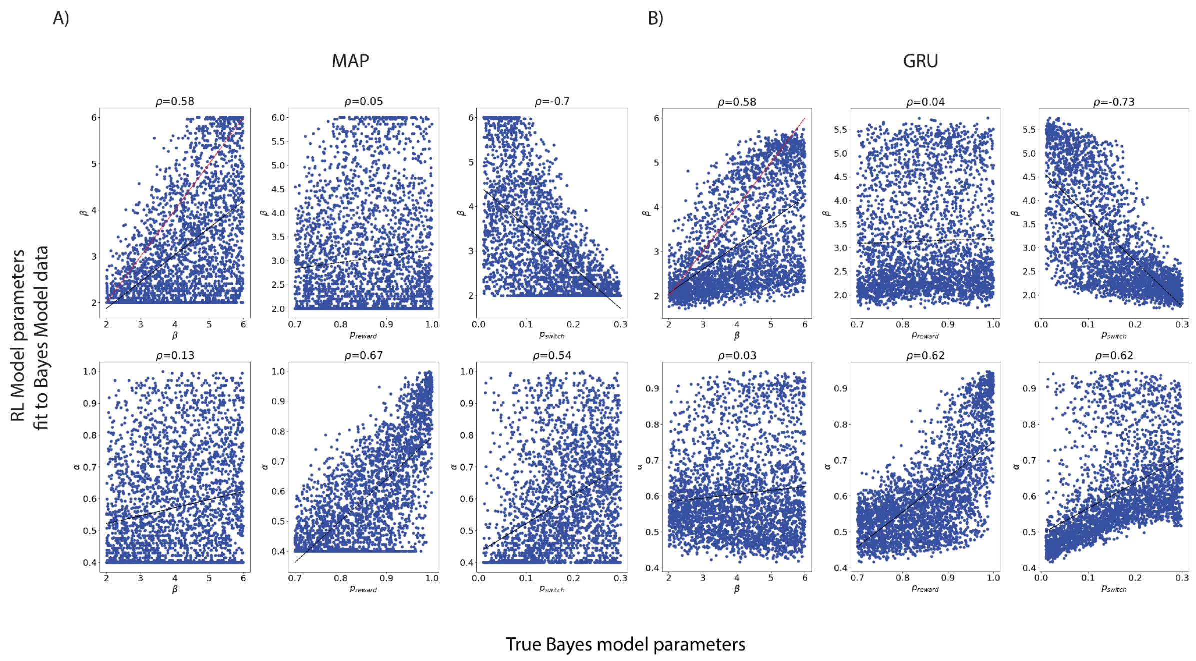

We repeated the same process in reverse (e.g. data simulated from an RL model, but fitting Bayesian model with MAP/network trained on the Bayesian model):

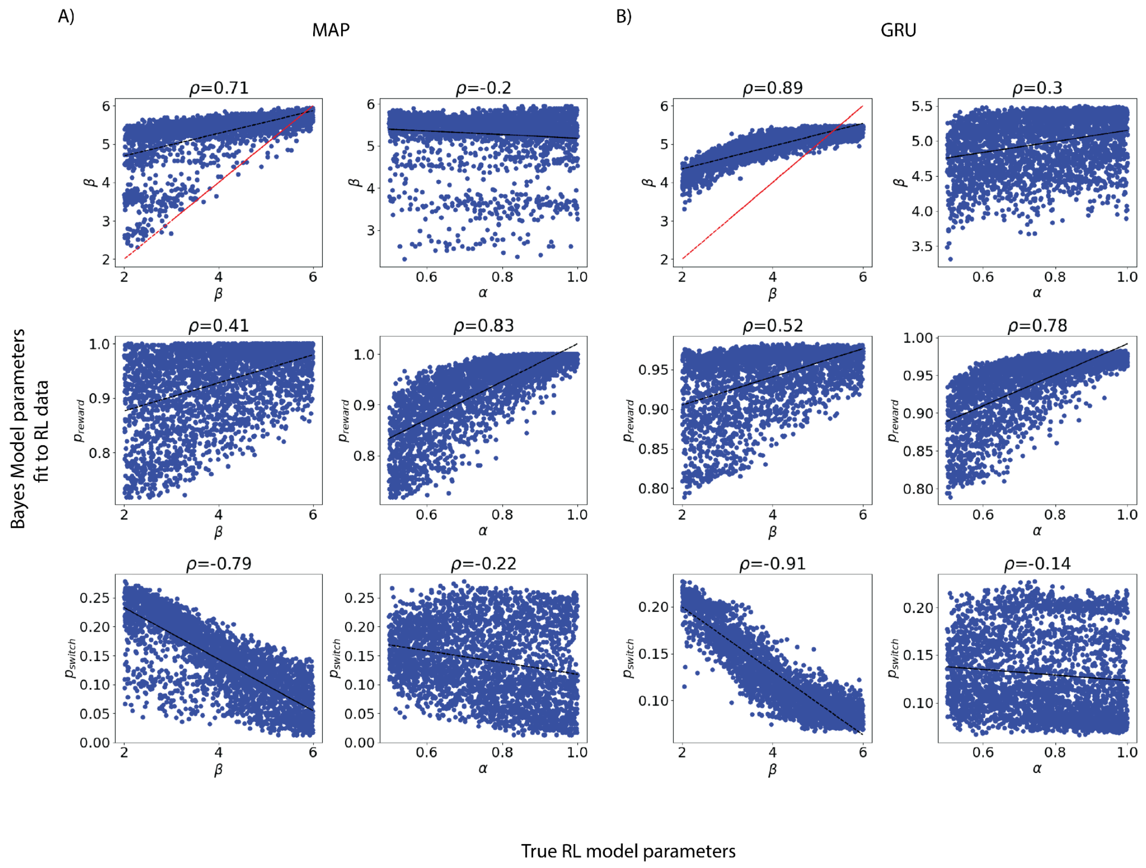

To more directly compare how our approach and a standard (MAP) approach react to model misspecification, we correlated parameter estimates obtained from MAP and GRU; high correlation would imply similarities in estimates between MAP and GRU, further confirming that ANNs are similarly impacted by model misspecification as traditional methods such as MAP.

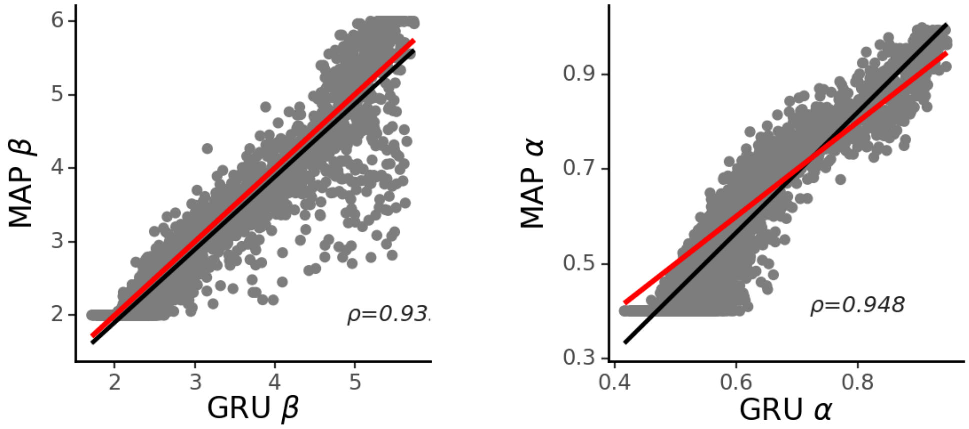

We also repeated the same comparison in reverse case (Simulate RL model, fit Bayes model parameters), again showing correlated estimates from the GRU and MAP (although with noisier MAP estimates):

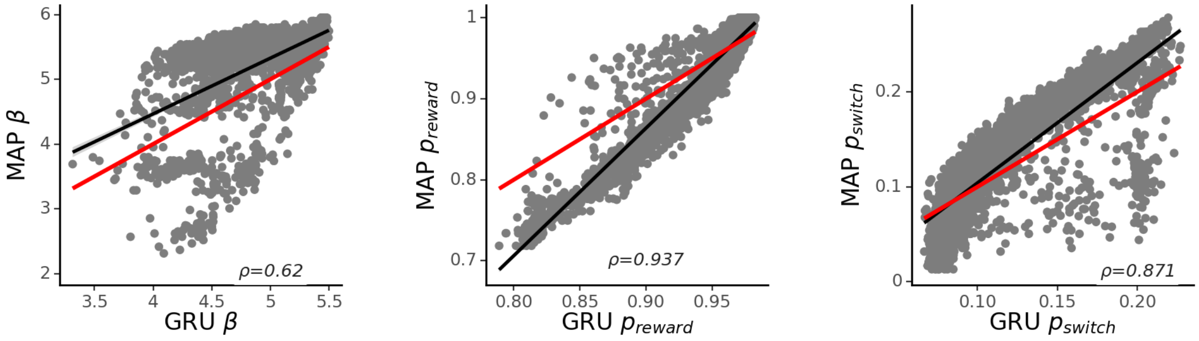

Reviewer #3:

Summary

I appreciate the authors' effort in revising key notions and claims in the paper as well as acknowledg ing a subset of the previous work in simulation-based inference (of which the authors' method is a part). I also appreciate the added robustness checks which raise the awareness for the real problems of model misspecification and out-of-distribution data that neural approximators face. Still, I believe there are some crucial misunderstandings regarding the relationship between the current approach and the vast sea of simulation-based inference methods available for solving the tasks at hand.

Major Points

The Integration of ABC and Neural Networks

Most recent neural approaches to simulation-based inference are not *integrated or combined* with ABC, as suggested, for example, by the new paragraph P3L95-100. Rather, they have been explicitly proposed and developed as an efficient alternative to ABC (e.g., Sokratous et al., 2023; Ghaderi-Kangavari et al., 2023; Cranmer et al., 2020; Radev et al., 2020; Greenberg et al., 2019; Goncalves et al., 2020) and this is independent of the particular neural architecture involved, be it a standard regression network, a normalizing flow (e.g., realized as an invertible network), or a diffusion model. The same goes for amortized model comparison (e.g., Radev et al., 2021). The authors' approach is a special case of (amortized) simulation-based inference with learned summary statistics (see below) and point estimates (instead of full posteriors or mean-variance estimates). Moreover, the recent works of Sokratous et al. (2023) and Ghaderi-Kangavari et al. (2023) should be clearly referenced as applications of neural networks for parameter estimation of cognitive model families.

Thank you for the comment. While we respectfully disagree with some of the reviewer's points, we feel that the disagreement is more at a semantic than deep level, and thus view the issue of framing as a minor aspect of our paper. Consequently, we have now modified the corresponding text in accordance with the reviewer's request to reflect their framing more closely (see e.g. lines 76-84). We have also cited the additional references.

The Role of Summary Statistics

The authors suggest repeatedly that their approach focuses on transforming data to parameters without the selection of summary statistics. This is misleading, as the summary statistics in the authors' lightweight regression approach are merely *implicit*. Consider, for instance, an arbitrary many-to-one recurrent neural network f trained to map sequences $x_{1:T} := x_1, x_2, \ldots, x_T$ to model parameters θ, $\hat{\theta} = f(x_{1:T})$. Clearly, the transformation realized by the network can always be simply decomposed into a learnable compression h (the many-to-one part) followed by a learnable projection g (the regression part) operation,

$$f(x_{1:T}) = g \circ h(x_{1:T}). \quad (1)$$

Thus, the projection cannot possibly be trained to generate the correct parameters estimates (e.g., the posterior mean in case of using the $L2$ loss or the MAP using standard weight regularization), unless the compression is a sufficient summary statistic for the conditional point quantity of interest. Sticking with the $L2$ loss and the posterior mean, the following must hold for correct point estimation,

$$\mathrm{E}\left[\theta \mid x_{1:T}\right] = \mathrm{E}\left[\theta \mid g(x_{1:T})\right]. \quad (2)$$

In other words, the fact that summary statistics are not considered does not mean that they are not there. Moreover, explicit (fixed or learned) summary statistics are a key proxy for detecting and rectifying the impact of model misspecification (e.g., Ghaderi-Kangavari et al., 2023; Frazier et al., 2020; Frazier and Drovandi, 2021; Schmitt et al., 2021; Huang et al., 2023).

Thanks for the comment. We of course agree with the reviewer that, indeed, our approach generates *implicit* internal summary statistics - as the reviewer mentions, this is self-evident within this type of method and we chose not to emphasize this point, since generating (implicit or explicit) summary statistics was not a goal of our approach. We nevertheless are happy to take the reviewer's suggestion to make this point explicit to the reader by adding “explicit” [implicit] when discussing the reviewer’s [our] methods, which differs from ours in its *explicit* goal of generating summary statistics (e.g. see lines 95-102, 328-330)

Uncertainty Quantification

The authors can easily extend their parameter estimation approach to a heteroskedastic regression which at least outputs the posterior variance along the posterior mean of the parameters (e.g., Kendall and Gal, 2017; Radev et al., 2019). The extension consists in minimizing the following loss

$$\mathcal{L} = \frac{1}{N}\sum_{i=1}^{N}\left[\frac{\log(\boldsymbol{\sigma}_i^2)}{2} + \frac{(\boldsymbol{\theta}_i - \boldsymbol{\mu}_i)^2}{2\boldsymbol{\sigma}_i^2}\right]$$

in expectation over the simulator outputs, where μ and σ are the outputs of the regression network. I see no technical barriers for enabling this straightforward extension, given the authors’ expertise in implementing the current workflows.

Thank you for the suggestion. We've added a section called "Uncertainty of Parameter Estimates" (lines 201-217) which presents results with the uncertainty estimates derived from incorporating evidential learning (as outlined by Amini et al.,2020) into our method.
